# A Catalog of Neutral and Deleterious Polymorphism in Yeast

**DOI:** 10.1371/journal.pgen.1000183

**Published:** 2008-08-29

**Authors:** Scott W. Doniger, Hyun Seok Kim, Devjanee Swain, Daniella Corcuera, Morgan Williams, Shiaw-Pyng Yang, Justin C. Fay

**Affiliations:** 1 Computational Biology Program, Washington University, St. Louis, Missouri, United States of America; 2 Molecular Genetics and Genomics Program, Washington University, St. Louis, Missouri, United States of America; 3 Biomedical Engineering Program, Washington University, St. Louis, Missouri, United States of America; 4 Spelman College, Atlanta, Georgia, United States of America; 5 Department of Genetics, Washington University, St. Louis, Missouri, United States of America; 6 Genome and Sequencing Center, Washington University, St. Louis, Missouri, United States of America; 7 Center for Genome Sciences, Washington University, St. Louis, Missouri, United States of America; University of Chicago, United States of America

## Abstract

The abundance and identity of functional variation segregating in natural populations is paramount to dissecting the molecular basis of quantitative traits as well as human genetic diseases. Genome sequencing of multiple organisms of the same species provides an efficient means of cataloging rearrangements, insertion, or deletion polymorphisms (InDels) and single-nucleotide polymorphisms (SNPs). While inbreeding depression and heterosis imply that a substantial amount of polymorphism is deleterious, distinguishing deleterious from neutral polymorphism remains a significant challenge. To identify deleterious and neutral DNA sequence variation within *Saccharomyces cerevisiae*, we sequenced the genome of a vineyard and oak tree strain and compared them to a reference genome. Among these three strains, 6% of the genome is variable, mostly attributable to variation in genome content that results from large InDels. Out of the 88,000 polymorphisms identified, 93% are SNPs and a small but significant fraction can be attributed to recent interspecific introgression and ectopic gene conversion. In comparison to the reference genome, there is substantial evidence for functional variation in gene content and structure that results from large InDels, frame-shifts, and polymorphic start and stop codons. Comparison of polymorphism to divergence reveals scant evidence for positive selection but an abundance of evidence for deleterious SNPs. We estimate that 12% of coding and 7% of noncoding SNPs are deleterious. Based on divergence among 11 yeast species, we identified 1,666 nonsynonymous SNPs that disrupt conserved amino acids and 1,863 noncoding SNPs that disrupt conserved noncoding motifs. The deleterious coding SNPs include those known to affect quantitative traits, and a subset of the deleterious noncoding SNPs occurs in the promoters of genes that show allele-specific expression, implying that some cis-regulatory SNPs are deleterious. Our results show that the genome sequences of both closely and distantly related species provide a means of identifying deleterious polymorphisms that disrupt functionally conserved coding and noncoding sequences.

## Author Summary

DNA sequence variation makes an important contribution to most traits that vary in natural populations. However, mapping mutations that underlie a trait of interest is a significant challenge. Genome sequencing of multiple organisms provides a complete list of DNA sequence differences responsible for any trait that differs among the organisms. Yet, distinguishing those DNA sequence variants that contribute to a trait from all other variants is not easy. Here, we sequence the genomes of two strains of yeast and, through comparisons with a reference genome, we catalog multiple types of DNA sequence variation among the three strains. Using a variety of comparative genomics methods, we show that a substantial fraction of DNA sequence variations has deleterious effects on fitness. Finally, we show that a subset of deleterious mutations is associated with changes in gene expression levels. Our results imply that comparative genomics methods will be a valuable approach to identifying DNA sequence changes underlying numerous traits of interest.

## Introduction

DNA sequence polymorphism makes a major contribution to phenotypic variation and provides a mean by which natural selection can lead to microevolutionary change and divergence between species. Since the first methods were developed to systematically survey DNA polymorphism within species and divergence between species [1], there has been a long-standing effort to identify and characterize this variation. Currently, genome sequences have been generated for a wide range of species and comparative genomic methods have identified coding and noncoding sequences that are functionally conserved across distantly related species [2]–[7], and characterized the phylogenetic distribution of these sequences, which is not always constant [8]–[13]. Recently, more closely related genomes have been sequenced in order to identify and characterize DNA polymorphism and divergence within functional and nonfunctional sequences [14]–[18]. Although the focus on differences between closely related species poses new challenges to comparative genomics methods, such as accounting for alignment and sequencing error, the main challenge lies in distinguishing polymorphisms with phenotypic and fitness consequences from those that are inconsequential.

A number of approaches have been developed to identify and characterize DNA polymorphism or divergence with positive or negative effects on fitness. Applications of these approaches have revealed that many aspects of DNA polymorphism and divergence can be explained by mutation and genetic drift, consistent with the neutral theory of molecular evolution [19]. Yet, two general observations indicate that adaptive changes within genomes are common. First, a reduction in levels of polymorphism in regions of low recombination is indicative of selective sweeps of advantageous alleles through a population and has been observed in a number of species [20]–[25]. Second, an excess of fixed differences relative to that expected based on polymorphism data is indicative of positive selection and has been observed for both coding and noncoding regions of the genome [26]–[30]. Although some methods are capable of identifying individual sites under positive selection [31]–[33], most statistical tests of neutrality only result in the identification of regions of the genome or genes that have been under selection. Thus, low resolution limits the ability of most methods to identify the molecular changes under positive selection and the phenotypic effects of these changes.

There is abundant evidence that deleterious mutations also make a significant contribution to phenotypic variation and DNA polymorphism [34],[35]. In diploid organisms, the pervasive effects of deleterious mutations are revealed by the decline in fitness as a function of inbreeding and an increase in fitness when outcrossed [34]. Based on the increase in child mortality and morbidity with inbreeding, it has been estimated that each human carries recessive deleterious mutations that if homozygous would result in premature death [36]. In *Drosophila melanogaster*, wild-caught flies carry deleterious mutations that result in a 60% average reduction in viability and an estimated 97% reduction in net fitness when made homozygous [37],[38]. Although many lethal or severely detrimental mutations are rare, persisting for 50–100 generations [39],[40], more weakly deleterious mutations may reach appreciable frequencies. For example, the frequency of null enzyme alleles is estimated to be just over 10^−3^ in flies [41] pine trees [42] and humans [43].

There are a number of estimates of the fraction of DNA polymorphism that is deleterious. Compared to the frequency distribution of allozymes expected from population genetic theory, there is a vast excess of low frequency alleles in both *Drosophila* and humans [44],[45]. This cannot be explained by a recent increase in population size, indicating that a substantial fraction, 20–40%, of nonsynonymous SNPs are deleterious [28],[46]. Methods based on conservation across species and protein structure have resulted in similar findings, leading to the estimate that each human carries on the order of 10^3^ deleterious nonsynonymous SNPs [46]–[50]. Despite the observation that 15–80% of sequences conserved between species are noncoding [51], the abundance of deleterious noncoding SNPs is not as well characterized.

Distinguishing deleterious SNPs from those that are neutral is a necessary but difficult step in identifying the molecular basis of quantitative traits and many diseases. Current methods based on protein structure and protein conservation across species show high false positive rates, 10–30% [48]. While additional structural and conservation data may improve the power of the methods, they are only applicable to predicting deleterious SNPs in protein coding sequences. Comparative genomics methods can identify sequences under purifying selection regardless of their function. However, distinguishing neutral and deleterious SNPs within conserved sequences requires single-base resolution of functional constraint and an inordinate number of genome sequences at an appropriate phylogenetic distance [52]. Single-base resolution of functional constraint may be attained by combining information from adjacent sites to both define the function of the sequence and predict whether a SNP disrupts that function. For example, previous work has shown that there is sufficient divergence among *S. cerevisiae* and two of its closest relatives to identify individual instances of conserved transcription factor binding sites [53]. Currently, with numerous fungal genomes to define constrained sequences and with numerous models of transcription factor binding sites [54],[55], genome-wide predictions of deleterious coding and noncoding SNPs is feasible.

As an initial investigation into cataloging whole-genome DNA polymorphism in *S. cerevisiae* and identifying the subset of variation with functional consequences, we sequenced the genome of two strains: M22, a strain isolated from a vineyard in Italy, and YPS163, a strain isolated from an oak tree in the United States of America [56]. We systematically cataloged sequence variation between these two strains and S288C, a laboratory strain for which there is a complete reference genome sequence, and compare this polymorphism to divergence from *S. paradoxus*, the closest known relative of *S. cerevisiae*. Combining both population genetics and comparative genomics methods, we find abundant evidence for deleterious coding and noncoding SNPs and we resolve a significant fraction of these differences to single polymorphic sites. Our results imply that comparative genomics can identify polymorphisms that underlie quantitative traits and human diseases.

## Results

### Genome Sequencing, Assembly, and Alignment

Two natural isolates of *S. cerevisiae*, M22 and YPS163, were sequenced by whole-genome shotgun and assembled using PCAP [57]. Forty thousand reads generated two-fold coverage of the genome and assemblies of 12 Mbp including gaps (Table 1). Excluding gaps, each assembly consisted of 10.7 Mbp of DNA, 88% of the size of the reference nuclear genome, S288C (Saccharomyces Genome Database).

**Table 1:** Sequence data and assembly.

Strain	Sequences			Contigs		Supercontigs		Assembly (Mbp)	
	Reads	Q20 Bases	Coverage	Number	N50	Number	N50	Bases	Coverage
M22	38,241	25,256,487	2.09236	4,481	2,479	1,695	14,234	10.7	12.1
YPS163	40,823	28,698,836	2.37754	3,752	3,067	1,072	19,581	10.7	11.9

Q20 bases are those with a Phred quality score of 20 or more. Coverage is the number of Q20 bases divided by the S288C nuclear genome size of 12.1 Mbp. N50 is median length in bp.

To identify DNA sequence polymorphism, we separately aligned the M22 and YPS163 assemblies to the S288C reference genome (see Text S1). Excluding gaps, the alignments include 11.4 Mbp of S288C sequence, 94% of the nuclear S288C genome. After combining the pairwise alignments, 8.3 Mbp of the alignments contain data from all three strains (Table 2).

**Table 2:** Sequences, SNPs and InDels within the combined alignments.

Alignment	Sites			Polymorphic sites	
	Bases	InDels	Gaps	SNPs	InDels
M22-S288C	1,367,559	71,913	1,703,911	5,621	499
YPS163-S288C	1,703,911	57,860	1,367,559	10,773	423
M22-YPS163-S288C	8,317,567	127,834	698,048	65,647	5,477
Total	11,389,037	257,607	3,769,518	82,041	6,399

SNPs have quality scores of 20 or more. Indels have quality scores of 40 or more within 2 bp of the indel and 50 or more for mononucleotide repeats.

### Genome Rearrangements

To identify any rearrangements between M22, YPS163 and the S288C reference genome, we mapped paired reads to the reference genome. Inversions or translocations should result in read-pairs inconsistent with the reference genome sequence. After removing single inconsistent read-pairs, which could be the result of chimeric clones, we found 5 inconsistent pairs in YPS163 that map to a single break and 11 inconsistent pairs in M22 that map to three breakpoints (Text S1 and Table S1). One of these rearrangements is a known reciprocal translocation between chromosome VIII and XVI that causes an increase in sulfite resistance in wine strains due to the creation of a new promoter for the sulfite membrane pump, *SSU1* [58],[59]. The other rearrangements all involve subtelomeric translocations and are not likely reciprocal since inconsistent read-pairs span breakpoints in only one direction. By PCR, we confirmed the reciprocal translocation in M22. We were unable to generate diagnostic PCR products for the other putative rearrangements due to the uncertainty in the breakpoints and repetitive sequences.

### DNA Polymorphism and Sequencing Errors

More than 88 thousand polymorphisms were identified within the combined genome alignments (Table 2). 93% of the polymorphisms are single nucleotide polymorphisms (SNPs) and 7% are insertion/deletion polymorphisms (InDels). However, out of 5.9% of bases found to differ among the three strains, 5.0% are the result of large (>100 bp) InDels and unaligned sequences (Table 3). Seventy-five of the large InDels, covering ∼2% of the alignments, are the result of transposable elements present in S288C but absent in M22 and/or YPS163 (Table S2).

**Table 3:** Summary of polymorphism data.

Category	Number of sites	Size (kb)	Coverage
Absent in S288C	29	71	0.6%
Absent in M22 and YPS163	27	261	2.3%
Large InDels (>100 bp)	119	238	2.1%
Small InDels (<100 bp)	6,280	21	0.2%
SNPs	82,042	82	0.7%
All	88,497	11,389	5.91%

The coverage is calculated using the 11.4 Mbp of aligned sequences. Sequences absent in M22 and YPS163 are based on shared gaps greater than 5 kb. Sequences absent in S288C are based on M22 and YPS163 sequence that match other yeast strains but not S288C.

Using a Phred quality score cutoff of 20 [60], a total of 393 SNPs and 2226 InDels are expected to be errors in the M22 and YPS163 assemblies. Using a quality score cutoff of 40, a total of 52 sequencing errors are expected in the two assemblies. However, 5,590 fewer SNPs passed this more stringent cutoff. For SNPs, a Phred quality ctuoff of 20 was used for most of the analysis and a cutoff of 40 was used for analysis of individual SNPs, e.g. distinguishing deleterious and neutral SNPs. To eliminate InDel errors, InDels were required to have Phred quality scores of 40 or more within two bp of the InDel and Phred quality scores of 50 or more within mononucleotide repeats.

### Strain-Specific Polymorphisms

**Figure 1:**
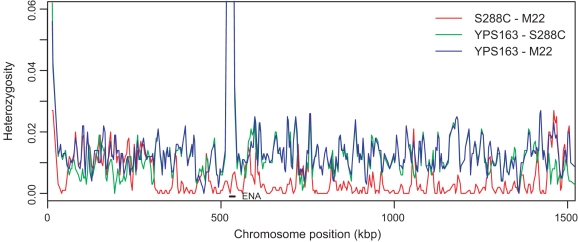
Variation in levels of heterozygosity across chromosome IV. Sliding windows of heterozygosity were obtained using 1000 synonymous sites and a step size of 500 sites for comparison of S288C and M22 (red), S288C and YPS163 (green), and M22 and YPS163 (blue). The highly polymorphic ENA locus is labeled by a black bar.

SNPs and InDels were classified into strain-specific variants for all cases where there were data from all three strains (Table S3). For both SNPs and InDels, YPS163 carries half of the strain-specific variants, M22 carries a third and the remainder are present in S288C. The large number of YPS163-specific alleles does not appear to be randomly distributed across the genome (Figure 1); in some regions, there are very few differences between M22 and S288C such that most variation is YPS163-specific. Similar mosaic patterns of variation were observed in whole-genome genotyping data and may be related to the hybrid origin of the S288C laboratory strain [61],[62].

### Introgression with *S. paradoxus*

**Figure 2:**
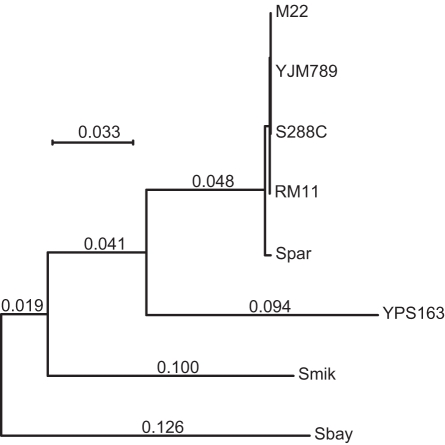
Maximum likelihood phylogeny of *EHD3* shows introgression of *S. paradoxus* into some strains of *S. cerevisiae*. *EHD3* homologs are from *S. paradoxus* (Spar), *S. mikatae* (Smik), *S. bayanus* (Sbay), and two other strains of *S. cerevisiae*, RM11 and YJM789. All nodes above *S. paradoxus* show 100% bootstrap support.

Using a sliding window of synonymous site diversity, the region with the highest rate of polymorphism (16.2%) occurs on chromosome IV (Figure 1). The high rate of diversity is not limited to a single gene. Six genes show a rate greater than 3.2%, the cutoff for the top 1% of windows: *ARO3* (4.6%), *EHD3* (4.8%), *KRS1* (15.1%), *ENA5* (16.4%), *ENA2* (3.9%), and *ENA1* (3.5%). The maximum likelihood tree of *EHD3* shows that all strains except YPS163 group with *S. paradoxus* (Figure 2). The difference between the gene tree and the species' known phylogenetic relationship, in which *S. paradoxus* is always an outgroup to strains of *S. cerevisiae*, indicates a recent transfer of the *S. paradoxus EHD3* allele into the common ancestor of most but not all strains of *S. cerevisiae*.

To examine the frequency of recent introgression between species and identify which genes have been introgressed across the genome, we used a phylogenetic analysis to examine genes showing high ratios of polymorphism within *S. cerevisiae* to divergence between *S. cerevisiae* and *S. paradoxus*. Out of 50 genes examined, 12 showed some phylogenetic evidence and 17 showed strong evidence of recent introgression (Text S1 and Dataset S1).

### Polymorphisms Caused by Ectopic Gene Conversion

Ectopic gene conversion has been found to occur among Ty elements [63], telomeric Y' elements [64] and a substantial number of duplicate genes [3],[65],[66]. Consistent with the effects of ectopic gene conversion, repetitive sequences show elevated rates of polymorphism but not divergence relative to synonymous sites (Table S4). To determine whether some polymorphisms are the result of ectopic gene conversion, the sequences flanking each SNP were compared to each other and to the rest of the genome. A total of 3816 SNPs reside within 25 bp of sequence that are repeated at least once in the genome. In 474 instances, the flanking sequences not only matched another sequence in the genome, but the same SNP was identified at that other position, making misassembly or misalignment unlikely. Of the 474 multicopy SNPs, 125 matched repeated sequences at a single locus, including the highly polymorphic genes *MUC1* (43 SNPs) and *NUM1* (30 SNPs). The remaining 349 cases involve SNPs within repeats found at two or more loci, including transposable elements (89), noncoding regions (62), telomeres (28) and a number of multigene families (Dataset S1).

### A Paucity of Evidence for Positive Selection

Comparison of polymorphism to divergence in *Drosophila* and other species has revealed pervasive evidence for positive selection. First, levels of polymorphisms are correlated with recombination but not divergence [20]–[25]. Second, the McDonald-Kreitman test [67] has shown elevated rates of nonsynonymous divergence across the genome [26]–[30].

To examine the correlation between neutral polymorphism and the rate of recombination, synonymous site diversity was measured in windows of 1000 synonymous sites with a step size of 500 sites. Based on 4527 regions with an average size of 4.5 kb, we found a weak but significant correlation between recombination and levels of polymorphism (P<10^−3^, Kendall's tau = 0.035, [68]).

Application of a McDonald-Kreitman style test to coding and noncoding regions revealed abundant evidence for negative selection against deleterious polymorphisms but no evidence for positive selection driving divergence between species. The ratio of nonsynonymous to synonymous polymorphism is significantly higher than that of divergence (likelihood ratio test, P<1×10^−299^, Table 4). Similarly, the ratio of polymorphism in conserved versus unconserved noncoding sites is significantly higher than that of divergence (likelihood ratio test, P<1×10^−21^, Table 4).

**Table 4:** Relative rates of polymorphism and divergence within coding and noncoding sequences.

		Coding			Noncoding		
		dN	dS	dN/dS	dC	dU	dC/dU
Polymorphism	S288C	0.0006	0.0033	0.19	0.0014	0.0023	0.62
	M22	0.0010	0.0058	0.17	0.0025	0.0039	0.66
	YPS163	0.0014	0.0112	0.12	0.0039	0.0066	0.60
	Total	0.0030	0.0203	0.15	0.0079	0.0127	0.62
Divergence	*S.paradoxus*	0.043	0.425	0.10	0.131	0.260	0.50

Divergence is between S288C and *S. paradoxus*. dN, dS, dC and dU are the rates of substitution in nonsynonymous, synonymous, conserved, and unconserved sites, respectively. Conserved and unconserved are defined by identity between *S. mikatae* and *S. bayanus*. Coding sequences contain 5.73×10^6^ nonsynonymous and 1.75×10^6^ synonymous sites. Noncoding sequences contain 1.16×10^6^ conserved and 1.12×10^6^ unconserved sites. All sites contain alignments from all three strains.

To test whether individual coding or noncoding regions have evolved under positive selection, we applied the same McDonald-Kreitman style test to 3834 coding and 1899 noncoding regions that contain four or more polymorphic sites. Within coding regions, 148 genes are significant (likelihood ratio test, P<0.01, uncorrected for multiple tests, Dataset S1). Yet, only four of the significant genes show evidence for positive rather than negative selection. Within noncoding regions, 31 regions are significant (likelihood ratio test, P<0.01, uncorrected for multiple tests, Dataset S1) and only six show evidence of positive selection.

### A Large Number of Deleterious Polymorphisms

The genome-wide McDonald-Kreitman style tests imply that a significant fraction of polymorphism is deleterious and will not contribute to divergence. To estimate the number and frequency of deleterious nonsynonymous SNPs, we estimated the number of neutral nonsynonymous SNPs from the ratio of nonsynonymous to synonymous divergence between species for those alignments containing sequence data from all three strains [46]. The observed relative to the expected number of nonsynonymous SNPs implies that over all three strains, 36% of nonsynonymous SNPs (12% of all coding SNPs) are deleterious (Table 5). Similarly, the observed relative to expected number of polymorphisms in conserved noncoding sequences implies that 19% of noncoding SNPs in conserved regions (7% of all noncoding SNPs) are deleterious. The relative frequency of deleterious coding and noncoding SNPs is similar. Of the nearly 7000 SNPs inferred to be deleterious, 21% lie in noncoding sequences and 23% of sites in the genome alignments are noncoding.

**Table 5:** Frequency of deleterious coding and noncoding SNPs.

Strain	Coding				Noncoding			
	Total		Deleterious		Total		Deleterious	
	Non	Syn	Number	Fraction	Con	Uncon	Number	Fraction
S288C	5,522	9,936	2,662	0.48	1,886	3,146	341	0.18
M22	4,177	7,383	1,760	0.42	2,409	3,509	565	0.23
YPS163	5,679	14,110	1,081	0.19	3,698	6,066	585	0.16
All three	15,378	31,429	5,503	0.36	7,993	12,721	1,491	0.19

The alignments and data are the same as those used in Table 4. The number of deleterious SNPs is the difference between the total and neutral number of SNPs. The number of neutral nonsynonymous SNPs is estimated by Non^*^dNpS/dSpN, where Non is the number of Nonsynonymous SNPs and dN, dS, pN, pS are nonsynonymous and synonymous divergences and polymorphism, respectively. Classes are nonsynonymous (non), synonymous (syn), conserved noncoding (con), unconserved noncoding (uncon). The deleterious fraction is the number of deleterious SNPs divided by the total number of SNPs in the class.

The estimated frequency of deleterious SNPs differs between strains. Because there is only a single unrooted tree for the three strains, each bi-allelic SNP can be assigned to a single lineage and strain-specific substitution rates can be estimated. Although most SNPs occur on the YPS163 lineage, YPS163 contains the smallest proportion of deleterious SNPs in both coding and noncoding sequences (Table 5). The proportion of nonsynonymous SNPs that are deleterious is significantly lower in YPS163 compared to either M22 or S288C (Fisher's exact test, P<1×10^−42^). The frequency of deleterious SNPs in M22 and S288C are not significantly different from one another. In contrast, the proportion of conserved noncoding SNPs that are deleterious is greater in M22 compared to either YPS163 or S288C (Fisher's exact test, P<0.001), the later two not being significantly different from one another.

### Distinguishing Deleterious from Neutral SNPs

Sequence conservation provides a mean of identifying functionally constrained sequences and deleterious SNPs that disrupt these sequences. However, distinguishing deleterious from neutral SNPs requires single-base resolution of functional constraint and thus a large set of distantly related species. To identify deleterious SNPs within coding and noncoding sequences, we examined conservation across distantly related fungi.

Starting with the genomes most closely related to *S. cerevisiae*, we selected four *sensu strictu Saccharomyces* species, *S. paradoxus*, *S. mikatae*, *S. kudriavzevii*, and *S. bayanus*, and six other hemiascomyetes species, *Candida glabrata*, *S. castelli*, *S. kluyveri*, *Kluyveromyces lactis*, *Ashbya gossypii*, and *Candida albicans*. Using alignments from 2046 genes, we found a median synonymous substitution rate of 18 substitutions per site. At this distance, single bases may still show identity across species by chance, but conservation of multiple sites, such as a single codon or transcription factor binding site, should be exceedingly rare.

**Figure 3:**
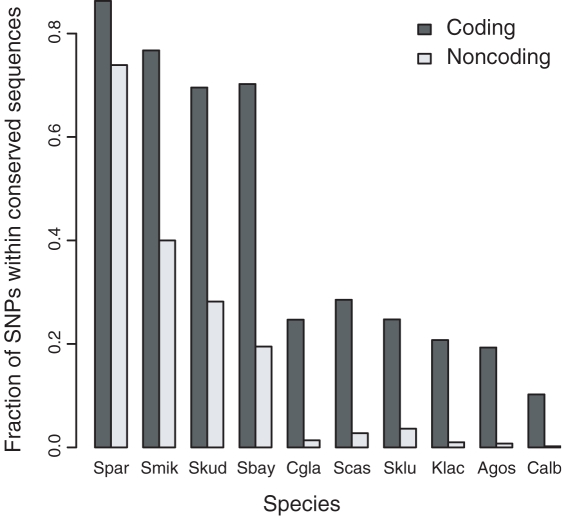
Conservation of SNPs containing coding and noncoding sequences in different yeast species. The fraction of *S. cerevisiae* SNPs in sequences conserved to *S. paradoxus* (Spar), *S. mikatae* (Smik), *S. kudriavzevii* (Skud), and *S. bayanus* (Sbay), *C. glabrata* (Cgla) *S. castelli* (Scas), *S. kluyveri* (Sklu), *K. lactis* (Klac), *A. gossypii* (Agos), and *C. albicans* (Calb). Homologous coding and noncoding sequences were identified by TBLASTX and BLASTN, respectively.

To generate an alignment for each coding and noncoding SNP, we used BLAST to search for homologous sequences within 100 bp of each SNP in each species. Relative to coding sequences, far fewer homologous noncoding sequences were identified (Figure 3). For coding sequences, 4,324 out of 15,454 (28%) high-quality, Phred quality score of 40 or more, nonsynonymous SNPs lie in sequences conserved in at least two other species outside of the *sensu strictu Saccharomyces* species. In contrast, only 813 out of 30,333 (2.7%) high-quality, noncoding SNPs met the same criteria.

Given a set of protein coding alignments, the SIFT algorithm uses the type of amino acid substitution in combination with average conservation across an alignment to determine whether a nonsynonymous SNP is deleterious [49]. SIFT predicted 970 (22%) of the nonsynonymous SNPs affect the function of the protein (SIFT score<0.05). However, it is difficult to estimate the error rate since SIFT scores depend on the average level of conservation within a protein, which differs for each protein and even within a single protein. For example, when applied to SNPs in alignments with fewer than two homologs outside of the *sensu strictu* species, SIFT predicted 2,841 (26%) of nonsynonymous SNPs affect protein function.

To incorporate phylogenetic distance into the likelihood of a SNP being deleterious, we compared the likelihood of a set of aligned sequences under a neutral and a conserved phylogenetic model. Under the neutral model the rate of nonsynonymous substitution within a codon is the same as the rate of synonymous substitution across the genome. Under the conserved model, the nonsynonymous rate within a codon is a fraction of the synonymous rate. Applying a likelihood ratio test to all high-quality SNPs in sequences with homologs in at least two species beyond the *sensu strictu Saccharomyces* species, we identified 1472 SNPs that disrupt significantly conserved codons (P<0.001), 34% of all nonsynonymous SNPs with distant homologs.

The deleterious SNPs are not randomly distributed. Out of the 1472 deleterious coding SNPs, there are many more deleterious SNPs in S288C (618) than in M22 (457) or YPS163 (401), similar to the overall frequency deleterious SNPs in coding sequences (Table 5). The 1472 SNPs occur in 1080 genes, five of which have seven or more deleterious SNPs: *ADH1*, *CDC47*, *FKS1*, *IMD4*, and *SSB1*. All five of these genes have paraologs in S288C and have percent identity that is often greater than 85%, implying that any changes in function caused by the SNPs may be buffered by paralogous genes. However, most deleterious SNPs are unlikely to be buffered: only 106 (7%) of the deleterious SNPs occur within sequences with paralogs that show greater than 85% identity within 100 bp of the SNP.

### Deleterious SNPs in Transcription Factor Binding Sites

Comparative genomics methods have been used to estimate that 34–43% of noncoding sequences are selectively constrained in yeast [53],[69]. However, less than 3% of noncoding SNPs lie in sequences that are conserved across distantly related yeast species. Thus, closely related species must be used to identify the 7% of all noncoding SNPs estimated to be deleterious (Table 5). Although there is not sufficient divergence to identify single bases under constraint, deleterious and neutral changes can be distinguished if they are known to occur within functionally conserved transcription factor binding sites, which constitute a significant fraction of conserved noncoding sequences [53],[69].

Previous studies have shown that there is sufficient divergence among the *sensu strictu Saccharomyces* species to reliably identify instances of conserved binding sites [53]. Further, methods have been developed to compare the likelihood of any nucleotide change under a binding site model and a neutral model in a phylogenetic context [8],[70]. To identify SNPs that disrupt conserved transcription factor binding sites, we used likelihood ratio tests to distinguish conserved transcription factor binding sites from neutrally evolving sequences and to distinguish neutral and deleterious SNPs within conserved binding sites.

To test each SNP, we used 422 models of transcription factor binding sites from various sources [54],[71] and 1,981,495 bp of aligned intergenic sequences that include at least three other *sensu strictu Saccharomyces* species. Out of 16,401 high-quality, Phred quality of 40 or more, noncoding SNPs examined, 2083 (13%) occur in sequences annotated as conserved binding sites (P<0.01), typically two conserved binding site predictions per SNP. For each SNP within a conserved binding site, we calculated the posterior probability of the SNP under a conserved binding site model and a model with loss along the SNP containing lineage [8]. For SNPs within multiple conserved binding site models, we calculated the average posterior probability weighted by the fit of the conserved model to the data. Compared to the conserved model, 1191 of the SNPs are twice as likely under a binding site loss model, suggesting that these SNPs introduce an unprefered nucleotide into a transcription factor binding site. The same analysis on shuffled noncoding alignments identified 597 SNPs that are twice as likely under the loss model, implying a false discovery rate of 50%. Using a more conservative cutoff for conserved binding sites (P<0.001), 383/636 SNPs are twice as likely under the loss model. The same analysis on shuffled alignments indicates a false discovery rate of 20% for identifying deleterious SNPs that result in loss of conserved transcription factor binding sites. Of these 1191 SNPs predicted to disrupt a conserved binding site, 761 (64%) are in positions that are identical across the *Saccharomyces sensu strictu* species. Only 13% of positions show both SNP alleles present in other species.

### Deleterious SNPs in Conserved Noncoding Motifs

In the previous section, we used models of transcription factor binding sites to identify deleterious SNPs. There are a number of concerns with this approach. First, most models of transcription factor binding sites are estimates of the true model and slight errors in these estimates are not accounted for in the binding site loss model [8]. Second, sequences bound by the same factor may evolve under different selective constraints, e.g. selection for strong or weak binding, resulting in a SNP being deleterious in one binding site but not another. Finally, identifying deleterious noncoding SNPs is predicated on a complete list of binding site models. One way of ameliorating these concerns is to combine motif finding with the identification of deleterious SNPs to avoid using published binding site models.

**Figure 4:**
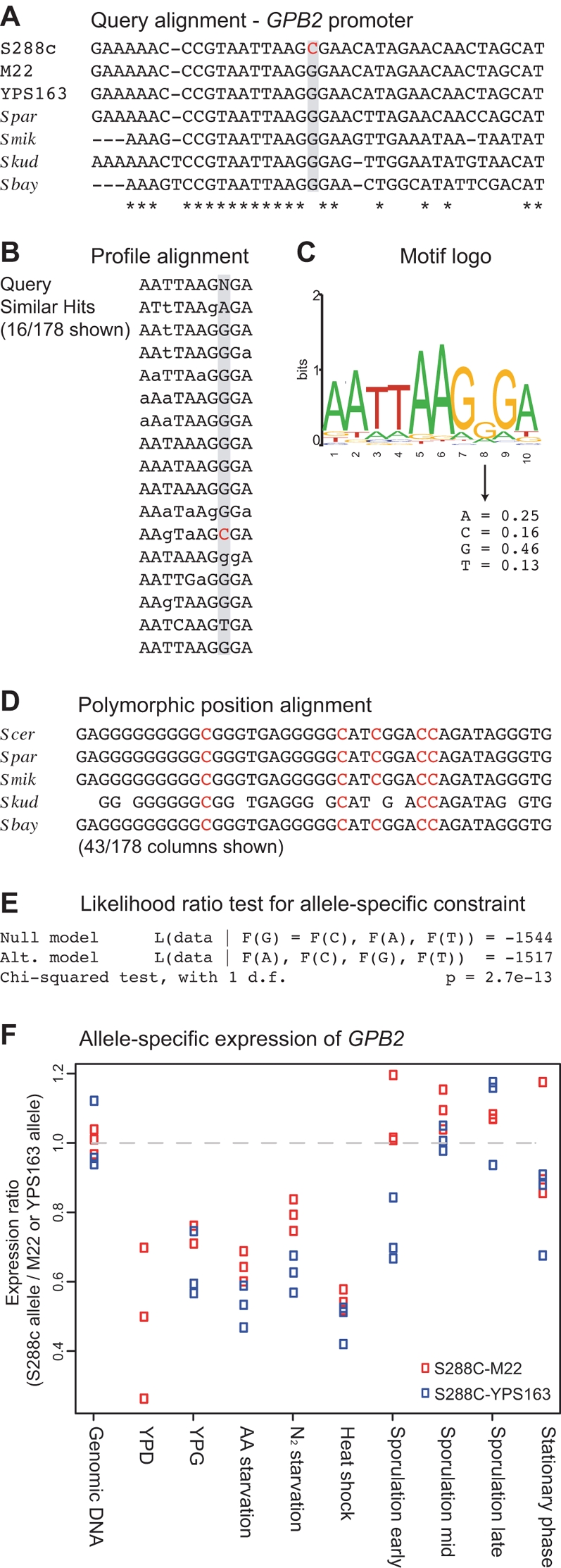
Identifying deleterious SNPs using Phylonet. Using a SNP in the promoter of *GPB2* as an example, a profile of conservation was generated from sequences adjacent to a SNP (A) and compared to all other noncoding profiles in the genome, ignoring the SNP containing column. Sixteen of the 178 profile alignments are shown with upper case letters indicating conservation across all species and instances of the derived SNP allele highlighted in red (B). The motif generated from these alignments is shown by a motif logo (C). Extracting the SNP containing positions from each profile alignment (D), a likelihood ratio test was used to determine whether the two SNP alleles were selectively equivalent to one another, measured by comparing the likelihood under equal base frequencies to the likelihood under a model where one allele is preferred over the other (E). Allele-specific expression of *GPB2* (F) shows that the S288C allele has reduced expression relative to M22 and YPS163 (P = 0.0027). The difference is found in YPD, YPG, amino acid (AA) starvation, nitrogen (N_2_) starvation and heat shock, which shows the maximum difference, 1.85-fold and 2.08-fold in comparison to M22 and YPS163, respectively.

Using the Phylonet algorithm, we identified conserved noncoding sequences similar to those flanking a noncoding SNP of interest. Phylonet is a motif finding algorithm that uses a BLAST-like method to search the genome for sequences with conservation profiles similar to that of a query profile [54]. To determine whether there is any preference between two SNP alleles, we modified Phylonet to mask the SNP position in the query alignment. The resulting list of profile alignments were then used to obtain unbiased estimates of the frequency of the two SNP alleles and determine whether they were significantly different from one another. By this method, the likelihood of a SNP being deleterious is different depending on whether it occurs in a weak or strong binding site and depending on the number of similarly conserved motifs. Figure 4 shows an example of this approach applied to a SNP in the promoter of *GPB2*, including the query alignment, the resulting profile alignments, the motif model and the likelihood ratio test for a significant difference in allele frequency.

Phylonet identified 4762 SNPs (30% of those tested) as occurring within multiple copy conserved motifs. The median number of alignments per motif was 36. HyPhy was used to implement a likelihood ratio test comparing the probability of the two SNP alleles being equivalent to each other or not. A total of 2452 and 1643 SNPs were significant at a low (P<0.01) and high (P<0.001) confidence cutoff, respectively. Application of the same method to shuffled alignments produced 191 and 104 SNPs, suggesting a false discovery rate of 7.7% and 6.2% for the low and high confidence cutoffs, respectively.

### Comparison of Deleterious SNP Predictions

**Figure 5:**
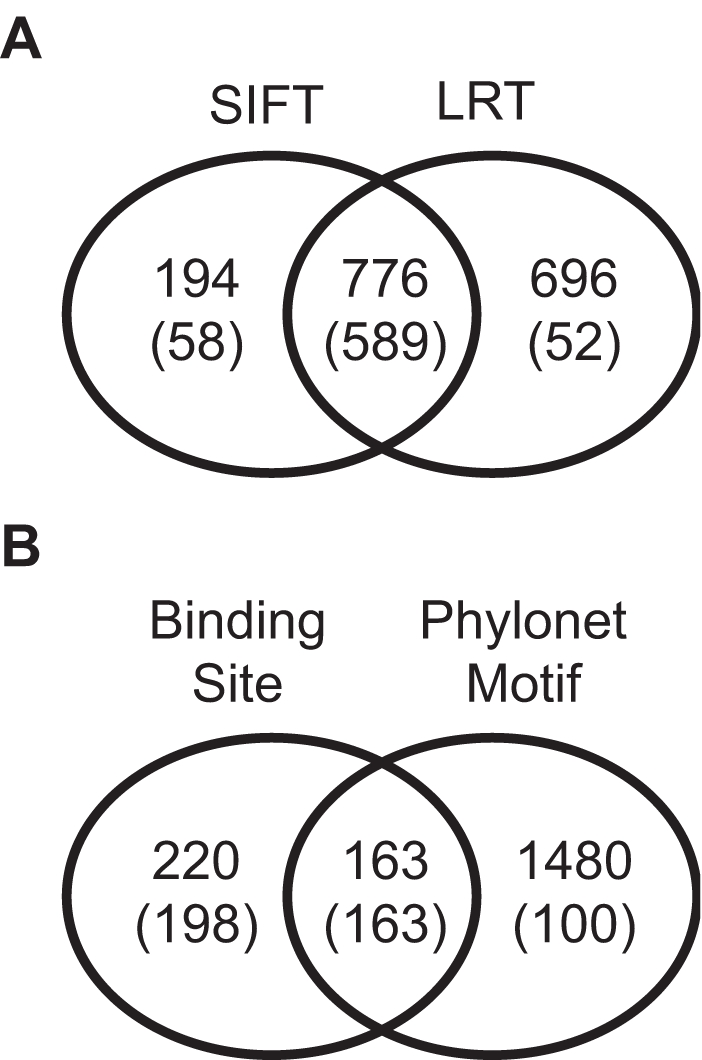
Comparison of deleterious SNPs predicted by different methods. The overlap between nonsynonymous SNPs predicted by the likelihood ratio test (LRT) and SIFT (A). The numbers in parentheses are for the subset of predictions within perfectly conserved amino acid positions. The overlap between noncoding SNPs predicted by the binding site model and the Phylonet motif model (B). The numbers in parentheses are for the subset of SNPs that occur in both conserved binding sites and conserved Phylonet motifs.

The overlap between deleterious nonsynonymous SNPs predicted by SIFT and the likelihood ratio test (LRT) is high, 47% of all SNPs identified (Figure 5). The overlap between the two methods is 84% for those SNPs that disrupt amino acids perfectly conserved across all species. Thus, the difference between the two methods is mostly due to constrained but not perfectly conserved amino acid positions.

The overlap between deleterious noncoding SNPs predicted by the binding site model and the Phylonet-based motif model is low, 9% (Figure 5). This is not entirely due to the much larger number of Phylonet predictions; the overlap is 17% using the 1191 predictions from the binding site model with the less stringent cutoff. Some of the difference between the two sets of predictions can be attributed to SNPs only tested by one of the two models. Out of 598 SNPs that occur in both conserved binding sites and conserved Phylonet motifs, 361 SNPs were predicted deleterious by the binding site model and 263 SNPs were predicted deleterious by the Phylonet model. The overlap between these predictions, 35%, is much higher, indicating that part of the difference between the two methods lies in identifying a significantly conserved binding site or motif. Compared to Phastcons, a method that doesn't rely on multi-copy conserved sequences, 45% of SNPs predicted by the binding site model and 12% of SNPs predicted by the Phylonet model were identified as significantly conserved sites by Phastcons using a posteriori probability cutoff of greater than 0.90 [51].

### Deleterious cis-Regulatory SNPs

SNPs that disrupt conserved noncoding sequences may often affect the regulation of an adjacent gene. To test whether changes in gene expression are associated with deleterious noncoding SNPs, we used allele-specific expression assays to measure the expression of 190 genes in hybrids of either M22, YPS163 or S288C. Nine of the predicted SNPs occur in divergently transcribed intergenic sequences and so expression of both genes was measured in order to query a total of 181 SNPs. Although four of the genes contain two deleterious SNP predictions and one gene contains three predictions, 60% of the SNPs tested were not confounded with any other noncoding SNP in the same intergenic sequence.

Based on measurements of allele-specific expression across nine environmental conditions, we found 51 differentially expressed genes at a 5% false discovery rate (Table 6 and Dataset S1). In most cases, allele-specific expression was found in multiple conditions. For example, *GPB2* showed allele-specific expression in five of the nine conditions (Figure 4F). The maximum difference in allele-specific expression observed across the 9 conditions ranged from 1.12-fold to 14.3-fold, with a median value of 1.47-fold. As a positive and negative control, we also measured allele-specific expression for *RME1*, which contains an InDel in its promoter known to affect expression and sporulation efficiency [72], and *RAS2*, which contains an InDel known to affect sporulation without a concomitant change in gene expression levels [73]. As expected, the S288C allele of *RME1* was expressed 2.7-fold higher than that of YPS163 after 12 hours in sporulation medium and no significant change in expression was found for *RAS2*.

**Table 6:** Deleterious SNPs associated with changes in gene expression.

SNP Class	Tested	Significant^5^	Significant (%)
Binding Site Model Only^1^	25	9	36
Phylonet Model Only^1^	56	23	41
Both Binding Site and Phylonet Model^2^	28	5	18
Weak Evidence^3^	34	6	18
No evidence^4^	38	8	21
Total	181	51	28

1 Deleterious SNPs predicted by either the binding site or Phylonet model but not both.

2 Deleterious SNPs predicted by both models.

3 SNPs with lower confidence predictions (0.001<P<0.01) by either the binding site or Phylonet model.

4 SNPs not predicted by the binding site or Phylonet model with low or high confidence.

5 Significant differences in the expression of adjacent genes at a 5% false discovery rate.

Out of the 51 SNPs associated with allele-specific expression, 73% (37/51) were predicted deleterious by the binding site or the Phylonet model. In 86% (32/37) of cases showing allele-specific expression, SNPs were only predicted deleterious by one of the two models. However, not all predictions were associated with allele-specific expression. Expression differences were found for 36% of SNPs predicted by the binding site model but not Phylonet and 41% of SNPs predicted by the Phylonet but not the binding site model. Surprisingly, only 18% of SNPs predicted by both models were found associated with changes in gene expression. No obvious differences were found for SNPs predicted by one method versus those predicted deleterious by both methods, e.g. the median Phastcons posteriori probability is 0.83 for SNPs predicted by both models versus 0.77 for SNPs predicted by only one of the two models.

In contrast to SNPs with binding site or Phylonet predictions, 21% of SNPs without any prediction were associated with allele-specific expression. We also tested 34 SNPs predicted deleterious at a lower confidence cutoff (0.001<P<0.01) by either model. While only 6/34 (18%) of the low confidence SNPs were associated with allele-specific expression, 2/3 low confidence SNPs predicted by both models were associated with changes in expression. In comparison to a method that identifies blocks of conserved sequences, only 26% of SNPs with sites predicted to be conserved by Phastcons [51], using a posteriori probability cutoff of greater than 0.90, were associated with allele-specific expression.

## Discussion

Genome sequencing of multiple organisms from the same species makes it possible to both catalog DNA polymorphism and identify variation with fitness and/or functional consequences. Using genome sequences of two strains of *S. cerevisiae*, we found variation in genome content, structure and sequence. Overall, there is substantial evidence for functional variation, based on disruption of sequences annotated in the reference genome, and deleterious variation, based on disruption of sequences conserved across other yeast species.

### Towards a Catalog of DNA Polymorphism

Using whole-genome sequence data, we identified multiple types of polymorphism, including variation in genome content, structure and sequence. Assuming no major skew in the allele frequency spectrum [74], ∼1% of these variants should represent rare alleles, less than 1% population frequency.

We identified four genome rearrangements and validated one reciprocal translocation that was previously characterized [58],[59]. In comparison, a single 32.5 kb inversion polymorphism on chromosome XIV and a translocation between chromosomes VI and X were identified by sequencing a clinical isolate, YJM789, to near completion [18]. The YJM789 translocation is likely the same VI to X rearrangement found in M22 (Table S1). Due to low coverage, it's not clear whether M22 or YPS163 contain the inversion.

Although genome coverage was low, we found variation in genome content by identifying sequences present in one strain but absent in one or more strains and by identifying insertions or deletions in genome alignments. A total of 261 kb of S288C sequences are present as gaps of greater than 5 kb in length in both the M22 and YPS163 assemblies. Another 273 kb of gaps are present in just M22 or YPS163, but not both. One of the M22-specific gaps spans a 7.9 kb region on chromosome I that was introgressed from *S. paradoxus* into YJM789 but not S288C [18]. This M22-specific gap can be explained by high divergence rather than variation in genome content; a reexamination of sequences in the M22 assembly but not present in the genome alignments includes one contig which does align to this region using a set of less stringent BLAST parameters. Finally, a number of strain-specific gaps, totaling 90 kb, overlapped an InDel defined by the other two strains.

We also found sequences present in M22 or YPS163 but absent from S288C. A total of 71 kb of sequence was absent from the complete S288C genome but present in more than one of the four other strain of *S. cerevisiae* with genome sequence data. This is likely an underestimate since a total of 323 kb of assembled M22 or YPS163 sequences were not included in the alignments. Many of these sequences are likely M22 and YPS163 duplications since they often matched S288C where other contigs were aligned with higher similarity. The estimates of variation in genome content presented in Table 3 are similar to those obtained from the comparison of YJM789 and S288C, where 49 kb of sequence was found absent in S288C and 276 kb of sequence was found absent in YJM789 [18].

When covered by a contig, insertions and deletions were detected. We found 238 kb of large InDels, greater than 100 bp in length, and 21 kb of small InDels. The majority of the large InDels represent transposable elements present in S288C but absent in both M22 and YPS163. In only a few cases were InDels greater than one kb not associated with a transposable element. These cases include *ENA1*, *FLO9* and *TFP1*, the two former genes being recently duplicated in S288C. Interestingly, the latter is a YPS613-specific deletion that corresponds exactly to the intein *VDE*, a site-specific endonuclease that shows similarity to the *HO* endonuclease [75].

As measured by the number of distinct segregating sites, SNPs and small InDels are the most abundant class of polymorphism (Table 3). Similar to the analysis of YJM789 [18], the ratio of SNPs to InDels is high, 12.8. However, within noncoding regions the ratio of SNPs to InDels, 6.0, is more similar to the overall ratio of 7.3, obtained from the sequencing of a human genome [76]. The difference between coding and noncoding ratios of SNPs to InDels is expected since most InDels result in frame-shifts. The higher frequency of InDels in noncoding sequences may contribute to the paucity of noncoding sequences conserved across distantly related yeast (Figure 3).

Overall, our results are similar to other whole-genome polymorphism surveys [18],[76]; most DNA sequence variations are SNPs followed by InDels and structural variation; but variation in genome structure, including large InDels, makes the largest contribution to total sequence differences. Although the relative importance of large structural variations, small InDels and SNPs is still an open question [77], each is likely to contain a subset that is functional and relevant to phenotype variation present in nature.

### Introgression

We found evidence of introgression of 29 genes from 16 different chromosomal regions of *S. paradoxus* into *S. cereviae* (Dataset S1). One of the regions on chromosome IV shows the highest rate of polymorphism in the genomes of these three strains, and includes the tandemly duplicated *ENA1*, *ENA2* and *ENA5* genes, P-type ATPases that transport sodium and lithium out of the cell [78]. The association between a quantitative trait locus for resistance to lithium chloride [79] and introgression into S288C and M22, but not the lithium sensitive YPS163 strain implies that the introgressed region may be responsible for differences in lithium sensitivity. Previous studies found introgression of a 12 kb region on chromosome I from *S. paradoxus* into YJM789 but not S288C [18] and introgression of a 23 kb subtelomeric segment of chromosome XIV from *S. cerevisiae* into *S. paradoxus* [80]. The latter region covers ten genes for which we also found evidence of introgression.

### Functional Polymorphism

A complete or nearly complete catalog of DNA polymorphism provides unprecedented insight into the genetic basis of phenotypic variation present in nature. A number of lines of evidence suggest that a significant fraction of the polymorphisms that we identified has phenotypic consequences. First, variation in genome content includes genes present in one strain but not another. Second, numerous SNPs or InDels alter gene length, either by changing the start or stop codon or by shifting the frame.

Combining variation in genome content due to unaligned sequences, insertions, deletions or duplications, we found 591 kb of variable sequence, 5.2% of the 11.4 Mbp of aligned genome sequences (Table 3). This includes 15 putative coding sequences that do not match the S288C genome but show similarity to known proteins (Table S5) and 54 genes annotated in S288C that lie within gaps of 5 kb or more in both the M22-S288C and YPS163-S288C alignments (Dataset S1), suggesting that these are unlikely to be explained by low-coverage.

The Saccharomyces Genome Database provides updated annotations of the S288C reference genome and includes a list of genes not in the systematic S288C sequence and genes known to vary in copy number among strains [81]. A total of 38 nuclear encoded genes are listed as not present in the systematic S288C sequence, including five genes that function in sucrose degradation (SUC genes), 14 genes involved in maltose metabolism (MAL genes), 10 genes involved in melibiose metabolism (MEL genes), along with *KHS1*, *STRP*, *RTM1*, *AWA1*, *TAT3*, *MPR1*, *BIO6*, *MATA1*, and *MATA2*. (The MAT genes are a consequence of sequencing a haploid *alpha* strain.) In comparison, we found sequences similar to *KHS1*, *AWA1*, and *MPR1* in M22 or YPS163. In addition to the SUC, MAL and MEL genes, *CUP1* and *ASP3* are known to vary in copy number among strains. *CUP1* varies in copy number between strains, resulting in substantial variation in copper resistance [82]. Although gaps in the assemblies make it difficult to estimate copy number in M22 and YPS163, there is significant variation in copper resistance among these strains [56]. All four copies of the *ASP3* gene lie within a 28 kb gap present in both M22 and YPS163. Previous work has implicated variation in asparaginase gene content to strain differences in the utilization of dipeptides as a nitrogen source [83].

The majority of large InDels, 83/119, involve transposable elements, primarily Ty1 and Ty2 elements of the Copia family (Table S2). One of the Ty1 insertions occurs at the 3' end of *HAP1*, significantly impairing its function [84]. The remaining Ty associated InDels occur within noncoding sequences (Dataset S1), one of which occurs upstream of the copper transporter *CTR3* and results in loss of copper-dependent transcriptional regulation [85]. Although the effects of the other Ty InDels are uncertain, eight of the genes adjacent to these InDels are differentially expressed among strains segregating these InDels [56]: *ATP1*, *MET17*, *GRX3*, *PHO12*, *POR1*, *RPS25A*, *RPT2*, *YDL038C*. Of the 17 InDels that are not related to Ty elements and that include protein coding sequences, 12 involve in-frame tandem repeats: *BBC1*, *BUD27*, *CHS5*, *DDR48*, *EGT2*, *FIT1*, *NUP159*, *SED1*, *SPA2*, *YFR016C*, *YIL169C*, *YMR317W*, some of which have been previously described [86].

In addition to variation in gene content, we found a large set of genes with significantly altered protein products, as measured by frame-shifting InDels and SNPs that disrupt or create start or stop codons. Although false positive InDels due to sequencing errors is a concern, 146/503 frame-shifting InDels are unlikely errors since they are either greater than 3 bp in length or are present in both M22 and YPS163 (Dataset S1). High quality SNPs, Phred quality cutoff of 40, result in the loss of 34 start codons, 31 stop codons and create 92 premature stop codons. In comparison to known variation in gene structure, the Saccharomyces Genome Database lists 9 cases where InDels or SNPs result in the split of a single gene into two in S288C, four of which are annotated as pseudogenes. In comparison to variation in gene structure in different yeast species, Kellis *et al*. [3] found 32 cases of two adjacent genes joined into a single gene in at least two other *Saccharomyces* species, and 210 and 330 cases of different conserved start and stop codons, respectively. Although there is some overlap between previous annotations and variation in M22, YPS163 and S288C, most variation is new: only 41/146 large or shared Indels and 22/157 of the polymorphic start/stop codons occur in genes previously identified as showing variation in gene structure.

The functional significance of variation in gene structure is not easily known. There are, however, a number of compelling examples. An 11 bp deletion in the aquaporin, *AQY2*, splits the gene into two open reading frames and affects water transport and freeze tolerance [87],[88]. YPS163 does not have the 11 bp deletion and shows significantly greater freeze tolerance compared to both M22 or S288C [56]. Another example is a large 3885 bp deletion of *ENA1* in M22 relative to S288C. *ENA1* encodes a P-type ATPase sodium, lithium transporter and a major effect quantiative trait locus for resistance to lithium chloride maps to the tandemly duplicated ENA gene cluster [79]. Finally, *MUC1* has the most frame-shifting InDels in the genome (10) and different *MUC1* alleles have been shown to affect biofilm formation [89]. *MUC1* also has the largest number of nonsynonymous SNPs in the genome, 43, excluding nonsynonymous changes resulting from frame-shifting InDels. The high levels of variation at these loci make positive selection a plausible explanation.

### The Frequency of Positive Selection

In contrast to other species, particularly *Drosophila* species, we found little evidence that positive selection has made a significant impact on polymorphism or divergence. In *Drosophila* species, there is a strong correlation between rates of recombination and levels of neutral variation [21],[24],[90],[91], consistent with selective sweeps. We found a significant but very weak correlation that only accounts for ∼3% of variation in levels of polymorphism. However, the lack of a strong correlation may be a consequence of the mating system since other species that both self-fertilize and outcross also show a weak correlation [17],[20]. Alternatively, the high rate of recombination in yeast, 0.34 cM/kb on average [92], may limit the effects of hitchhiking to closely linked sites.

In *Drosophila* species, McDonald-Kreitman style tests have indicated that adaptive substitutions are common in both coding and noncoding sequences [26]–[30]. We found no evidence of positive selection either in individual genes or across the genome. Although the power to detect positive selection on individual genes is limited, there is no lack of power in the analysis of the combined data from multiple gene regions. One explanation for the lack of evidence for positive selection is an abundance of deleterious polymorphisms [28]. However, the abundance of deleterious polymorphisms is similar in *S. cerevisiae* and *D. melanogaster* (see below). The absence of evidence for positive selection in both inbred and outcrossing species of *Arabidopsis* suggests that special considerations may be needed to interpret the *Drosophila* rather than the yeast data [93].

### The Frequency of Deleterious Polymorphism

Our estimate of the proportion of nonsynonymous SNPs that are deleterious, 36%, is similar to estimates from *D. melanogaster*, 27% [28], and Humans, 20–38% [46]–[48],[50]. We also estimated that 19% of SNPs within conserved noncoding sequences are deleterious (Table 5). Since there is just as much conserved noncoding as coding sequence in most eukaryotic genomes, our results imply that noncoding sequences carry a significant fraction of deleterious SNPs present within the genome.

The frequency of deleterious SNPs differs among strains. One explanation is differences in selective constraint, caused by either differences in effective population size or differences in the selective regime [94]. Consistent with this explanation, S288C, which has recently been maintained in the near absence of natural selection in the laboratory, shows the highest proportion of deleterious to neutral SNPs in coding sequences (Table 5). Interestingly, M22 shows the highest proportion of deleterious SNPs in noncoding sequences and has been hypothesized to have gone through a recent population bottleneck with other vineyard strains of yeast [95]. The discrepancy between which strain has the most deleterious coding or noncoding SNPs may be the result of differences in the strength of selection on coding and noncoding sequences or differences between the genome histories of S288C and M22. In YPS163, the high rate of synonymous polymorphism combined with the low rate of deleterious SNPs implies that its genome experienced a larger effective population size than that of M22 and S288C.

### Distinguishing Deleterious and Neutral SNPs

Using a likelihood ratio test, we identified 1472 nonsynonymous SNPs that are deleterious. The likelihood ratio test differs from SIFT and other heuristic methods based on protein structure or sequence conservation [47],[49],[50]. Without a neutral expectation, heuristic approaches can have a high rate of false positives depending on the data to which they are applied [96]. We used synonymous sites as a neutral expectation. Although this approach may be quite sensitive, it may also identify SNPs that occur in positions that are slightly but not absolutely conserved, as a result of either weak or episodic selection. However, the predictions made by the likelihood ratio test and SIFT are quite similar for nonsynonymous SNPs that disrupt perfectly conserved amino acid positions (Figure 5).

Distinguishing deleterious and neutral SNPs in noncoding sequences is much more difficult than in coding sequences. First, even though 34–43% of noncoding sequences are selectively constrained [53],[69], only 3% of noncoding SNPs lie in sites that are conserved to distantly related species. The difference between conservation of coding and noncoding sequences (Figure 3) can be attributed to differences in the level and type of constraints in coding and noncoding sequences and to differences in the sensitivity of BLASTN versus BLASTP. Two aspects of divergence that likely differ between coding and noncoding sequences are compensatory changes, as hypothesized by the binding site turnover model [97], and InDels, which are much more common in noncoding sequences.

A number of methods have been developed to identify conserved noncoding sequences, e.g. [51],[98],[99]. However, closely related species that are readily aligned do not provide single-base resolution of selective constraint, i.e. single nucleotide sites are often expected to be identical in all species by chance. Using conservation of sequences adjacent to a SNP of interest, we used two a binding site model and a model based on Phylonet motifs to identify 1863 deleterious noncoding SNPs. Although the overlap between the two sets of predictions is low, much of this can be attributed to the low overlap between significantly conserved binding sites and significantly conserved Phylonet motifs (Figure 5). Regardless of the method, compensatory changes and binding site turnover [8] place substantial constraints on the power of comparative methods to identify all deleterious noncoding SNPs.

### Phenotypic Consequences of Deleterious SNPs

The phenotypic effects of some deleterious SNPs may be large. Out of eleven quantitative traits that have been mapped to single nucleotide sites, eight alter amino acids that are significantly conserved and are easily identified by the likelihood ratio test implemented in this study (Table 7). The test could not be applied to the quantitative trait nucleotide in *END3* since it occurs in a repetitive sequence and only closely related *sensu strictu* homologs were identified even after removing BLAST filters. Of the 1472 deleterious nonsynonymous SNPs, 393 have a likelihood ratio test P-value within the range of the cases listed in Table 7. This suggests that there is a large set of nonsynonymous SNPs with effects that could be as large as major effect quantitative trait nucleotides. The two quantitative trait nucleotides in noncoding sequences were both InDels and so the SNP-based binding site and Phylonet methods were not applied.

**Table 7:** Conservation of quantitative trait nucleotides.

Gene	SNP	LRT P-value	Phenotype	Reference
*ASP1*	D142H	7.9E-10	Acetic acid production	Marullo et al. 2007
*CYS4*	I123N	3.9E-09	Multi-drug sensitivity	Kim and Fay 2007
*END3*	S258N	NA	High temperature growth	Sinha et al. 2006
*GPA1*	S469I	9.7E-09	Cell elongation, gene regulation	Yvert et al. 2003; Nogami et al. 2007
*MKT1*	G30D	2.5E-08	Sporulation efficiency, High temperature growth	Deutschbauer et al. 2005; Sinha et al. 2006
*MLH1*	D761G	4.5E-07	Mismatch repair	Heck et al. 2006
*PHO84*	L259P	3.2E-08	Drug sensitivity	Perlstein et al. 2007
*PMS1*	R818K	1.1E-06	Mismatch repair	Heck et al. 2006
*TAO3*	E1493Q	2.9E-06	Sporulation efficiency	Deutschbauer et al. 2005
*RAS2*	A[-9]-	NA	Sporulation efficiency	Ben-Ari et al. 2006
*RME1*	-[-308]A	NA	Sporulation efficiency	Deutschbauer et al. 2005

LRT P-value is from alignments of distantly related homologs, no distant homologs were identified for the *END3* SNP. Promoter polymorphisms are indicated by position relative to the start codon in brackets, dash indicates deletion. The likelihood ratio test is not applicable to InDels.

Assessing the phenotypic effects of deleterious noncoding SNPs is more difficult. First, the majority of deleterious SNPs occur upstream of genes with no detectable allele-specific differences in expression. It is possible that some of the deleterious SNPs affect temporal aspects of gene regulation or disrupt sequences with functions other than gene regulation. For example, polymorphism in the 3' UTR of *RHO2* contributes to a high temperature growth phenotype but shows no detectable effect on *RHO2* mRNA expression levels [100]. Similarly, an InDel upstream of *RAS2* affects sporulation but shows no effect on *RAS2* mRNA expression level [73]. Another difficulty with assessing the effects of deleterious noncoding SNPs is that genetic variation in gene expression is abundant and much of it may be neutral [101],[102]. Consistent with these observations we found that 16% of SNPs associated with changes in gene expression were not predicted to be deleterious by any model (Table 6). However, 73% of the genes showing allele-specific expression contained deleterious SNPs upstream of their coding sequences, implying that a significant fraction of genetic variation in gene expression levels may be caused by deleterious cis-regulatory SNPs.

### Conclusions

The ability to sequence multiple organisms of the same species has the potential to revolutionize the analysis of quantitative traits and human diseases. Realizing this potential depends on our ability to link phenotypes to genotypes without being limited by recombination. Our analysis of DNA polymorphisms among three strains of yeast outline two general approaches to identifying candidate genotypes for any given phenotype. First, candidates can be selected from DNA polymorphisms that disrupt sequences that have been experimentally annotated, e.g. frame-shift and nonsense polymorphisms. Second, candidates can be selected from DNA polymorphisms that are deleterious and disrupt evolutionarily conserved coding or noncoding sequences. Although not all traits of interest may be caused by polymorphisms that disrupt annotated and/or conserved sequences, the abundance of candidates present in the genomes of three strains of yeast implies that they may impact a considerable number of traits. Our results show that probabilistic evolutionary models can be used to distinguish deleterious and neutral SNPs in both coding and noncoding sequences, an important step in identifying SNPs that underlie quantitative traits and human diseases.

## Materials and Methods

### Sequencing and Assembly

DNA was extracted from rho^−^ derivatives of *S. cerevisiae* strains YPS163, isolated from an Oak tree in Pennsylvania, and M22, isolated from a vineyard in Italy. Rho- strains were obtained by growth in minimal medium with 25 ug/ml ethidium bromide [103]. A genomic DNA library was made using the plasmid pOTw13 and sheared DNA with an average insert size of 3.8 kb. Sequencing was carried out in Washington University's Genome and Sequencing Center and reads were deposited into NCBI's trace archive (TI:2017509004-2017433010).

Genome assemblies were generated using PCAP [57]. Because of the low coverage, many singlet reads were not included in the assembled contigs. After eliminating unplaced reads of less than 500 bp, more than 50% low quality bases and those designated as repeats, 1686 unplaced reads from M22 and 990 from YPS163 were added to the assembly. Genome assemblies were deposited into GenBank (Accession ABPC00000000 and ABPD00000000).

### Genome Rearrangements

Genome rearrangements were identified by paired reads with unique but inconsistent matches to the reference genome. Unique matches were defined as those with a BLAST E-value less than 1.0e-90 and no secondary hit greater than 1.0e-50. Out of 16,074 read-pairs from M22 and 18,381 from YPS163, 37 YPS163 pairs and 27 M22 pairs were inconsistent with the reference genome either because they matched different chromosomes or matched greater than 20 kbp apart from one another on the same chromosome.

### Genome Alignments and DNA Polymorphism

Contigs from both M22 and YPS163 were aligned to the S288C reference genome using BLAST. Alignments were subsequently filter to ensure one-to-one alignment for both genome alignments: M22-S288C and YPS163-S288C. The two genome alignments were combined using S288C as a reference. Variation in genome content was identified by sequences not present in the genome alignments. SNPs and InDels were identified within the genome alignments. A more detailed description of methods and results can be found in Supporting Information (Text S1). A tally of synonymous, nonsynonymous and noncoding SNPs in each gene and noncoding sequence can be found in Supporting Information (Dataset S3).

### Polymorphism Relative to Recombination

Recombination rates were obtained from genetic mapping data [81] and Spo11 mediated mapping of double strand breaks [104]. A polynomial fit of genetic to physical distance was obtained for each chromosome using the loess function in the statistical software package R. The recombination rate for each interval of interest was obtained by calculating the slope of the fitted relationship over the interval. Since both the genetic mapping data and the double strand break data gave similar correlation coefficients with rates of polymorphism, only the genetic mapping data was used.

### McDonald-Kreitman Style Tests

Modified McDonald-Kreitman tests [67] were carried out using HyPhy [105]. This enabled the use of realistic nucleotide (HKY85) and codon (MG94xHKY85) substitution models and accurately accounted for multiple substitutions at the same site. For genome-wide comparisons, sequences were concatenated and a likelihood ratio test was used to determine whether constraints on polymorphism were significantly different from constraints on divergence for the polymorphism tree (M22, YPS163 and S288C) and the divergence tree (S288C and *S. paradoxus*). The two trees were simultaneously maximized and shared all parameters except those measuring selective constraints. For coding sequences, selective constraint was measured by the ratio of nonsynonymous to the synonymous substitution rate. For noncoding sequences, constraint was measured by the ratio of the substitution rate in conserved and unconserved noncoding sequences. To avoid bias, conserved noncoding sequences were defined by sequences identical in *S. mikatae*, *S. kudriavzevii* and *S. bayanus* or identical any two of the three species if one sequence was missing. Unconserved noncoding sequences were defined as the complement of the conserved sequences. Transposable elements and other noncoding genes were eliminated from the analysis. The likelihood ratio test was also applied to each coding and noncoding region individually.

### Number of Deleterious SNPs

The fraction of coding or noncoding SNPs that are deleterious was estimated by the difference between constraint estimated from polymorphism data and constraint estimated from divergence data [46]. In the absence of deleterious polymorphisms, constraint on coding sequences within species, pN/pS, should be the same as constraint on coding sequences between species, dN/dS, where dN, dS, pN, pS are the rate of nonsynonymous and synonymous divergences and polymorphism, respectively. The fraction of nonsynonymous SNPs that are deleterious was estimated by the ratio of these two constraints dNpS/dSpN. The expected number of deleterious SNPs was estimated by the fraction of deleterious SNPs multiplied by the observed number of SNPs. Similar calculations were made for noncoding SNPs.

### Deleterious SNPs in Conserved Sequences

A total of 11,075 nonsynonymous and 16,164 noncoding SNPs have a Phred quality score of 40 or more, excluding those in sequences within overlapping annotations. Homologs in 10 yeast genomes were identified using TBLASTX (word size = 3 amino acids, E-value<1e-10, filter = seg, qframe = 1) for coding SNPs and BLASTN (word size = 6, filter = dust, E-value<1e-10) for noncoding SNPs. For nonsynonymous SNPs we used the codon containing the SNP along with 33 codons upstream and downstream and for noncoding SNPs we used 100 bp upstream and downstream. Multiple alignments were generated by combining the ungapped BLAST alignments using S288C as a reference.

Deleterious SNPs were identified by a likelihood ratio test. For coding sequences we compared the likelihood of the codon containing the SNP under two models: dN equal to dS and dN less than dS, where dN and dS are the nonsynonymous and synonymous substitution rate respectively. A dS of 18 substitutions per site was obtained from the median synonymous substitution rate of 2046 genes using a set phylogeny [106]. dN was from the codon of interest. Significance was examined by comparing the likelihood ratio of the null and the alternative model, which has one additional parameter (selective constraint), to a Chi-squared distribution with one degree of freedom. The results are in Supporting Dataset S2.

### SNPs in Transcription Factor Binding Sites

Using 125 transcription factor binding site models from chromatin immunoprecipitation data [71], an NDT80 model from the literature [107], and 296 models predicted by the Phylonet algorithm [54], we tested each SNP that occurred in an *S. cerevisiae* sequence with a log-odds score greater than 2 for a match to the binding site model versus background [108]. By this criteria, a total of 125,520 tests were conducted for 16,164 high quality SNPs, Phred quality score cutoff of 40, for which there were alignments with fewer than 50% of gapped positions and with at least three other *sensu strictu Saccharomyces* species besides *S. cerevisiae*. To identify binding sites conserved across species, we calculated the likelihood under a neutral and conserved binding site model [8]. To identify SNPs that disrupt conserved binding sites, we added the strain containing the alternative SNP allele to the alignment and calculated the likelihood of the data under a conserved binding site model to one with loss along the lineage containing the derived allele. When multiple conserved binding sites were predicted for a single SNP, we calculated the average posterior probability of loss based on the five models with the best fit, weighted by the fit of each model to the data, where the fit is measured by the likelihood ratio of the conserved model relative to a neutral model.

### Deleterious SNPs Identified using Phylonet

The Phylonet algorithm is described in [54]. Three changes were made to the algorithm to identify sequences that show constraints similar to a query sequence containing a SNP. First, we masked the SNP position in the query so that the resulting alignments are unbiased with respect to the allele at the SNP position. Second, only motifs overlapping the SNP position were included in the analysis, those matching flanking sequences were discarded. Third, we eliminated those alignments inconsistent with the best motif model. Alignments were eliminated as follows. Using the algorithm's profile clustering method, all high scoring pairs were combined to find the best possible motif using the average log-likelihood ratio score. Each alignment was then scored again using only the positions within the boundary of the best motif and insignificant alignments were discarded. This step helped eliminate spurious sites that were high scoring alignments based on similarity to the query in sequences adjacent to the discovered motif and SNP of interest. In comparison, the original Phylonet algorithm uses all alignment scores to determine which overlapping profile alignments to generate predicted motifs from and can generate multiple motifs from a single query.

### Allele-Specific Expression

Allele-specific expression was measured in nine different environmental conditions: rich medium, glycerol, amino acid starvation, nitrogen starvation, heat shock, stationary phase, and three time points of sporulation. For rich medium, cells from an overnight YPD (1% yeast extract, 2% peptone, 2% dextrose) culture were diluted in fresh YPD and sampled after eight hours of growth. For glycerol, cells from an overnight YPD culture were diluted into YPG (2% glycerol, 2% peptone, 2% dextrose) and sampled after eight hours of growth. For amino acid starvation, cells from a mid-log phase culture of YPD were resuspended in minimal medium (0.67% yeast nitrogen base with ammonium sulfate, 2% glucose) and sampled after 45 minutes. For nitrogen starvation, cells from a mid-log phase YPD culture were resuspended in 2% dextrose with 0.17% yeast nitrogen base without ammonium sulfate and sampled after 8.5 hours of growth. For heat shock, cells from a mid-log phase YPD culture growing at 20C were transfered to 37C for 20 minutes and then sampled. For stationary phase, a YPD culture was grown for 54 hours and then sampled. For sporulation, cells from an overnight YPA (1% yeast, 2% peptone, 1% potassium acetate) culture were resuspended in 1% potassium acetate and sampled after 4, 8 and 12 hours. For the S288C-M22 hybrid, samples were taken at 8, 12 and 20 hours to account for the slower rate at which tetrads formed compared to the M22-YPS163 and S288C-YPS163 hybrids.

RNA was extracted using the Qiagen RNEasy kit from the nine environmental conditions for three biological replicates of each hybrid strain. cDNA was synthesized for each RNA sample using a poly-T primer and the Qiagen Omniscript RT kit. Genomic DNA was extracted from each hybrid for a reference. The samples were diluted to a concentration of 2.5 ng/ul and quantitatively genotyped using the Sequenom MASSArray method following the manufacturer's protocol (Sequenom, San Diego, CA). The allele frequencies of each SNP were obtained from the Sequenom allele frequency data report.

Significant differences in allele-specific expression were identified using an analysis of variance on the ratio of alleles across all conditions. Both cDNA and DNA samples were included in the analysis of variance. The same results were obtained if cDNA samples were analyzed alone after normalization to the DNA ratios. For the 86% of the cases where there was informative data from two hybrids, a term for the hybrid effect was included in the ANOVA model. The significance of was estimated by permutation resampling of the expression ratios, shuffling the column labels 20,000 times. A P-value cutoff of 0.019 corresponds to a false discovery rate of 5% (Dataset S1).

## Supporting Information

Table S1 Rearrangements identified by read-pairs inconsistent with the S288C reference genome.
(0.12 MB XLS)

Table S2 Summary of Insertion/deletion polymorphism greater than 100 bp in length.
(0.11 MB XLS)

Table S3 Strain-specific variation.
(0.11 MB XLS)

Table S4 Rate of polymorphism in *S. cerevisiae* compared to divergence between *S. cerevisiae* and *S. paradoxus* in different classes of sites.
(0.12 MB XLS)

Table S5 M22 and YPS163 sequences that do not align to the S288C genome.
(0.08 MB XLS)

Text S1 Supplemental results.
(0.17 MB PDF)

Dataset S1 Tabulated results.
(0.44 MB XLS)

Dataset S2 Genome polymorphism data.
(11.32 MB XLS)

Dataset S3 Deleterious coding and noncoding SNPs.
(3.33 MB XLS)
