# Supplementary material for: A Catalog of Neutral and Deleterious Polymorphism in Yeast
Source: PLoS Genet. 2008 Aug 29;4(8):e1000183. doi: 10.1371/journal.pgen.1000183 (PMC2515631; doi:10.1371/journal.pgen.1000183)
Supplement: Text S1 — Supplemental results. (0.17 MB PDF) [file pgen.1000183.s006.pdf]

SUPPORTING TEXT S1

Genome Alignments

M22 and YPS163 were separately aligned to the S288C reference genome using the highest scoring gapped alignment obtained by BLAST. Because orthology is not easily determined for duplicated sequences, we distinguished alignments containing duplicated and unique sequences (Table 1). For M22 and YPS163, 10% and 7% of the sequences in the assembly matched multiple sequences in the S288C genome, respectively. A similar proportion of the S288C genome matched more than one contig per assembly. Excluding all sequences with multiple alignments, roughly 10 Mbp or 80% of the S288C nuclear genome was unambiguously aligned to each strain (Table 1).

Table 1. Mega-bases in pair-wise genome alignments.

Strain	Query (M22 and YPS163)			Subject (S288C)		
	Duplicated	Unique	Total	Duplicated	Unique	Total
M22	1.07	9.44	10.51	1.20	9.16	10.36
YPS163	0.74	9.77	10.51	0.81	9.49	10.30

Query refers to M22 or YPS163 contigs.

Subject refers to the S288C reference genome.

Many alignments were partially redundant, containing the same S288C, M22, or YPS163 sequence as another alignment. Alignments can overlap with one another for a variety of reasons (Figure 1)[1]. First, overlapping contigs or supercontigs can occur when there is not sufficient overlap to assemble them. Second, overlapping alignments within the same contig can result from insertion/deletion (InDel) polymorphism flanked by direct repeats or polymorphic tandem duplications.

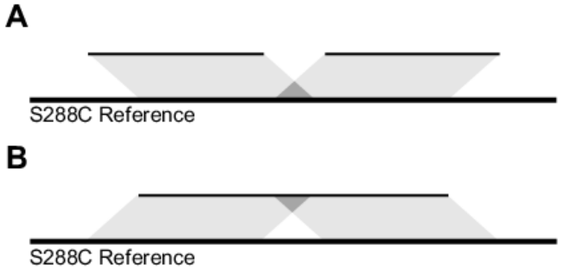

Figure 1. Overlapping alignments can result from unassembled contigs or insertions or deletions flanked by repeats. (A) Unassembled contigs (thin black lines) aligned to overlapping positions in the S288C reference genome (thick black line). Aligned regions are shown by gray. (B) The same contig can be aligned to multiple positions in the reference genome when there is an InDel flanked by a short repeat.

Roughly half of the overlapping supercontigs aligned to subtelomeric regions, known to contain many duplicated sequences [2, 3], or to the ribosomal RNA gene cluster, present at ~100 copies per cell on chromosome XII [4]. Consistent with unassembled supercontigs, those that did not align to

subtelomeric regions or the rDNA cluster overlap by only a small amount, ~100 bp (Table 2). We also identified 386 and 417 overlapping alignments that flanked gaps within supercontigs in M22 and YPS163, respectively (Table 2). To remove overlapping alignments between supercontigs and between adjacent contigs within supercontigs, we used the alignment with the fewest number of differences between the two sequences.

Table 2. Summary of overlapping alignments.

Strain	Supercontig overlap					Contig overlap**				
	Number			Length (bp)		Gap in query	Overlap of subject or query		Overlap of both	
	Total	Subtelo.	rDNA	Total	Median*	Number	Number	Length***	Number	Length***
M22	1682	139	872	813,921	71	386	217	8	82	26
YPS163	868	50	356	409,137	104	417	172	6	77	27

* The median excludes overlapping alignments within subtelomeric regions and rDNA.

** Contig overlap is divided into either gaps in the query contig (M22 and YPS163), overlapping query (M22 or YPS163), overlapping subject (S288C), or both.

*** Length is the median of the overlap.

Overlap within the same contig is expected when there is an insertion/deletion polymorphism flanked by direct repeats or a polymorphic tandem duplication (Figure 1). We identified 217 (M22) and 172 (YPS163) insertions/deletions (InDels) from alignments with overlapping query (M22 or YPS163) or subject (S288C) sequences. The InDels were flanked by direct but not always perfect repeats, typically 6-8 bp in length. Overlapping alignments from the same contig were eliminated by creating an insertion or deletion within one of the sequences.

Methods: Each supercontig was aligned to the S288C genome using WU-BLASTN 2.0MP (Gish, W. (1996-2004), http://blast.wustl.edu) with parameters: -hspsepsmax 40000 -M 1 -N -3 -Q 5 -R 1 -E 1e-20 -hspmax 10000 -gapS2 500 -gapW 64 -span1. The scoring parameters were chosen to optimize the search for sequence similarity of 99% (Gish, W. (1996-2004) http://blast.wustl.edu), and alignments with percent identity below 90% were not considered. A second, less stringent set of parameters was used to define duplicated sequences. These parameters were optimized for 90% sequence similarity and are the same as the first set except: -M 2 -N -3 -Q 10 -R 2 -span2. Under a variety of conditions the top scoring reciprocal BLAST hit need not be the orthologous sequence. Thus, sequences with unique alignments were distinguished from those that could be aligned to multiple sequences. A sequence was considered uniquely aligned to the reference genome if it aligned to no other sequence in the reference genome and no other query sequence aligned to the same position in the reference genome, using the less stringent BLAST parameters described above.

The BLAST alignments do not guarantee each base is present only once. Alignments contained either overlapping query sequences, overlapping subject sequences or both. Overlap occurred between high scoring pairs from the same contig, high scoring pairs from different contigs within the same supercontig, and between different supercontigs. These were all handled in a similar way. If two alignments contained an overlapping subject or overlapping query sequence, we used the alignment with fewer differences in the region of overlap and removed the redundant part of the other alignment. In the case of subject (S288C) overlap, if the two alignments came from a single contig and the unaligned query sequence between the two alignments contained no Ns, we used the alignment with fewer differences in the region of overlap and we replaced the redundant alignment with a gap in the subject sequence. In the case of query overlap (M22 or YPS163), we used the alignment with fewer differences in the region of overlap and we replaced the redundant alignment with a gap in the query sequence. In the case of both query and subject overlap, we simply chose one of the two alignments

and removed the redundant part from the other alignment.

The M22-S288C and YPS163-S288C alignments were combined into a multi-strain alignment based on the S288C reference. Insertion/deletions (InDels) at the same location were combined without considering the sequences within the gap. A total of 8.3 Mbp of sequence was aligned between all three strains, 9.7 Mbp was aligned between M22 and S288C and 10.0 Mbp was aligned between YPS163 and S288C. Overall, 2.2% of the alignments were gapped. The combined alignments are available upon request from the corresponding author.

S. paradoxus **alignment**

A total of 162,186 reads from *S. paradoxus* strain NRRL Y-17217 were download from the NCBI trace archive. PCAP produced a 11,860,950 bp assembly of 332 supercontigs and 792 contigs, with a median contig size of 60,817 bp. The *S. paradoxus* genome was aligned to S288C and combined with *S. cerevisiae* alignments using the same methods used to align and combine strains except that less stringent BLAST parameters were used: -M 5 -N -5 -Q 18 -R 3.

Variation in Genome Content

Large insertions and deletions can result in variation in genome content. There are two cases in which variation in genome content can be detected based on sequences not present in the genome alignments. In one case, sequences in M22 or YPS163 align to sequences in either RM11 [5] or YJM789 [6], ensuring that they are yeast sequences, but do not align to the S288C genome. In another case, sequences in the S288C genome do not align to M22 or YPS163. In both cases, the most likely explanation is a strain-specific deletion rather than a more complex micro-rearrangements or rapid sequence divergence.

A total of 16 YPS163 sequences (38 kb) and 13 M22 sequences (33 kb) were found to align to either RM11 or YJM789 but not S288C. Of these, five did not match proteins in the Saccharomyces Genome Database (SGD) [7] and eight showed similarity to hypothetical proteins or proteins of unknown function present in S288C or another fungal genome. The remaining 16 sequences have similarity to proteins of either a known or putative function (Table S4). These include sequences with similarity to genes that have been isolated from strains other than S288C, such as a foam-forming gene *AWA1* from a sake strain [8], a killer toxin *KHR1* [9], and a gene involved in regulation of maltose fermentation *MAL64* from *S. pastorianus* [10], an allopolyploid of *S. cerevisiae* and *S. bayanus* [11].

Because of low coverage, many gaps are expected to be present in the M22 and YPS163 assemblies. However, gaps greater than 5 kb in length are not expected given the average read length, number of reads and assuming a uniform distribution of reads across the genome [12]. Thus, cloning biases are not accounted for. A total of 57 gaps greater than 5 kb in length were identified in either the YPS163 or M22 alignments (Dataset S1). Twenty-seven of the gaps, covering 261 kb, were present in both the M22 and YPS163 alignments, suggesting that the gaps are not the result of low coverage. Many of the gaps included telomeric sequences (13), transposable elements (30), or occurred in subtelomeric regions of the genome (22). In some cases, the shared gaps are quite large and cover named genes. For example, a 29 kb gap on the tip of chromosome X includes *PAU1*, *FSP2*, *HXT9* and *HXT8*, a 29 kb gap next to the ribosomal gene cluster on chromosome XII includes four tandemly duplicated asparaginases, *ASP3-1*, *ASP3-2*, *ASP3-3*, and *ASP3-4* , and a 16 kb gap on the tip of chromosome XVI includes *ERR2* and *HSP32*. The largest gap present in only one of the two strains, YPS163, is 26 kb and includes three genes *NUP116*, *CSM3* and *ERB1*, flanked by two full length Ty1 transposons. Large gaps in YPS163 were also found at the tandemly duplicated sodium pumps *ENA1-*

ENA2 (6.4 kb) and the glucose transporters *HXT3-HXT6* (7.1 kb).

Methods: Sequences absent from the S288C genome were assessed by those sequences of 500 bp or more that were not present in the genome alignments. For M22, there were 60 sequences totaling 114 kb that were not present in the alignment with S288C. For YPS163, there were 87 sequences totaling 210 kb that were not present in the alignment with S288C. Many of these sequences were from parts of contigs that did align. Using the same BLAST parameters, many of the partial sequences aligned to S288C at a different position than the original contig, typically a duplicated sequence or a transposable element, suggesting chimeric clones, misassembly, or complex genomic variation. A total of 16 YPS163 and 13 M22 sequences did not return any BLAST hits to S288C but showed greater than 90% identify to either RM11 or YJM789.

Sequencing Errors

Based on the frequency distribution of Phred quality scores, a total of 1,484 and 1,135 sequencing errors are expected in the M22 and YPS163 assemblies, respectively, using a quality score cutoff of 20 [13]. The majority or errors (85%) are expected to be insertions or deletions, often in short mononucleotide repeats [13]. Thus, a total of 393 SNP and 2226 InDel errors are expected in the M22 and YPS163 assemblies.

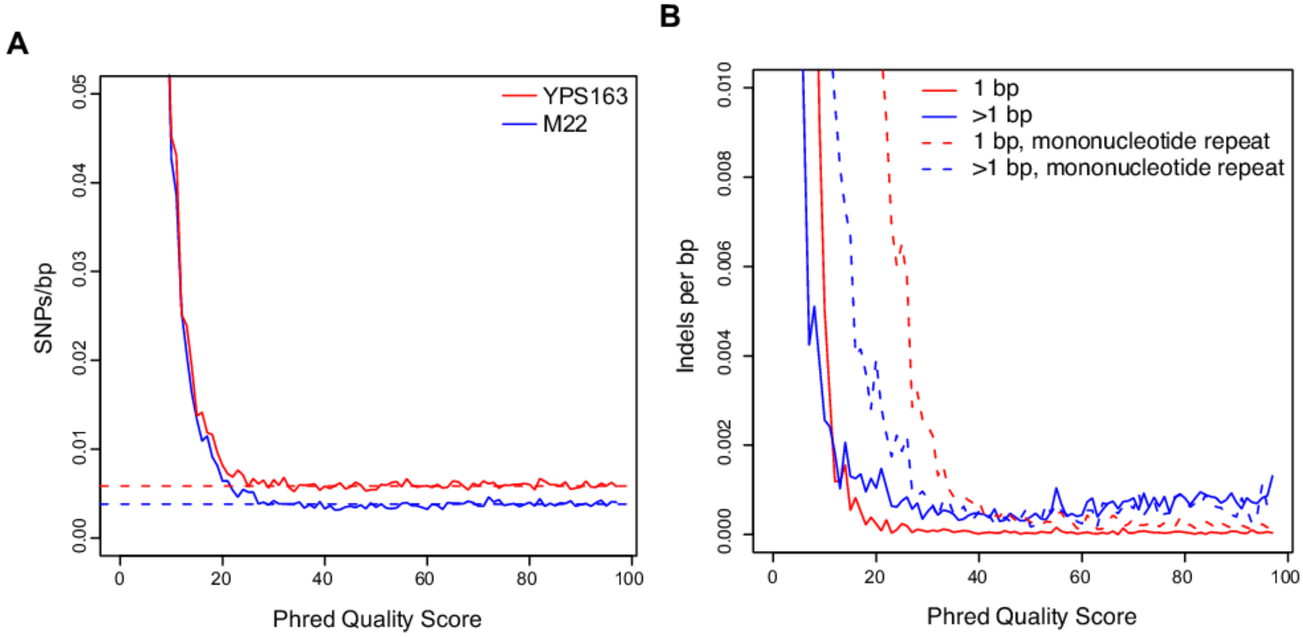

Figure 2. The rate of polymorphism for SNPs (A) and InDels (B) as a function of Phred quality scores. The average rate of polymorphism based on SNPs with quality scores over 50 is shown by the dotted line for M22 (blue) and YPS163 (red). The Phred quality of adjacent bases was used for deletions relative to the reference genome. For InDels within mononucleotide repeats, the minimum Phred quality score within the repeat was used. M22 and YPS163 InDels were combined and classified based on whether they were single-base (red) or multiple-base (blue) InDels and whether they occurred within mononucleotide repeats (solid line) or not (dashed line).

To examine SNP and InDel error rates empirically, we compared the rate of SNPs and InDels as a function of Phred quality score (Figure 2). Using the rate of SNPs with quality scores greater than 50 as the true rate, the estimated number of SNP errors in both assemblies is 340 at a Phred quality cutoff of 20. Estimating the rate of InDel errors is more difficult since the quality score of an InDel must be obtained from adjacent bases [14] and depends on whether the InDel occurs in a mononucleotide repeats or not (Figure 2).

Insertion and Deletion Polymorphism

Insertion/deletion (InDel) polymorphism was tabulated from the combined M22-YPS163-S288C genome alignments. There were 119 large InDels, greater than 100 bp in length, covering a total of 238 kb (Table S2 and Dataset S1). Most of these InDels (94/119) represented sequences present in the S288C genome but absent in either M22 (33), YPS163 (34) or both (27). The remaining InDels represented sequences absent in the reference genome but present in M22 (14), YPS163 (6) or both (5). The majority (74/94) of sequences present in the reference genome but absent in either M22 or YPS163 are associated with either solo LTRs or full length transposable elements (TEs), which include *GAG* and *POL* genes flanked by long terminal repeats (LTRs). InDels associated with the gain or loss of transposable elements account for a significant fraction of the 331 annotated elements within the S288C genome [15]; 36/51 full length TEs are polymorphic and 39/280 solo LTRs are polymorphic, suggesting most full length transpositions have occurred after the divergence of the strains. Many of these longer insertions are full length transposable elements in the Copia (Ty1, Ty2, Ty4) and Gypsy (Ty3) family of retrotransposons [15].

Nineteen of the large InDels contain coding regions. Out of the 19, 16 are in-frame and 10 of these insertions are tandem repeats. The three InDels that are not in-frame include a 283 bp M22-specific sequence within *ABM1*, required for normal microtubule organization [16], an S288C-specific sequence that includes the 3' end of the *FLO9* gene, a member of a family of cell-surface adhesion proteins involved in flocculation [17], and a S288C-specific duplication of *ENA1*, a P-type ATPase sodium pump that varies in the number of tandem repeats among strains [18, 19].

The majority of InDels are small, less than 10 bp (Figure 3). Out of the 6,280 InDels less than 100 bp in length, 960 occurred within coding sequences and 503 resulted in frame-shifts (Dataset S1). Many of the frame-shifts are not likely significant as they occur in dubious (184) or hypothetical (111) open reading frames, or within 100 bp of the stop codon (157). Of the 208 frame-shifts in characterized genes, 86 are unlikely to be sequencing errors as they are either present in both M22 and YPS163 or are longer than one bp in length. Excluding multiple InDels within the same gene that shift out and back into frame (46), that are within 100 bp of the start or stop codon (28), or that are flanked by low quality sequence (7), five genes with InDels remain: *AQY2*, *BUD4*, *ECM33*, *GAT1*, and *MSS11*. A small number of genes contain six or more frame-shifting InDels that shifted out and back into frame resulting in significant variation in the amino acid sequence: *MUC1*, *SRP40*, *MSS11*, *OCT1*, *BUD14* and *PTK1*. Interestingly, many of the genes with significant protein changes function in budding (*BUD4*, *BUD14*, *ECM33*) or invasive growth (*MSS11*, *MUC1*).

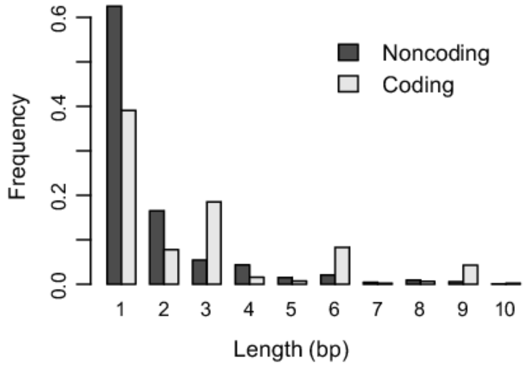

Figure 3. Distribution of insertion/deletion length in coding and noncoding sequences. Noncoding deletions and insertions have an average size of 3.25 and 3.19 bp.

Single Nucleotide Polymorphism

A total of 32,210 noncoding, 32,469 nonsynonymous, and 16,294 synonymous SNPs were identified. Within coding sequences, 1068 SNPs were not classified due to overlapping reading frames or the presence of three alleles in the three strains. Of the high quality coding SNPs, Phred quality score of 40 or more, that occurred in non-overlapping genes, 26 eliminated the start codon, 29 eliminated the stop codon and 71 created a new stop codon, in most instances more than 100 bp from the S288C stop codon. Over half of these SNPs resulting in changes in protein length occurred in genes with known functions (Dataset S1).

Introgression

To identify genes that have introgressed between *S. cerevisiae* and *S. paradoxus*, we compared the ratio of polymorphism to divergence. Across the genome, the median ratio of polymorphic sites to fixed differences is 0.047. Sixty genes showed a ratio of polymorphism to divergence greater than 0.30, many with a ratio close to one. From the 50 genes that we were able to generate suitable alignments that include either *S. mikatae* or *S. bayanus* as an outgroup. Seventeen genes showed strong evidence of recent introgression, based on the complete discordance between the gene trees and species trees across the entire gene, 12 showed some evidence for introgression, based on heterogeneity in the phylogenetic relationship, 9 showed evidence for gene conversion between paralogous *S. cerevisiae* genes and 12 showed no discordance between the gene tree and the species tree. Out of 13 genes showing complete introgression across the entire sequences, i.e. no phylogenetic heterogeneity, 12 showed greater than 97% bootstrap support for the relevant nodes.

The 17 genes showing strong evidence of introgression occur within seven regions (Dataset S1). One region on chromosome XIV includes 8 contiguous genes, 10 if genes with some evidence are included, spanning *AAD14* to YNL321. Interestingly, *AAD14* is at the telomeric end of this region and there are closely related paralogs of *AAD14* and its adjacent gene, *THI12*, on five other *S. cerevisiae* chromosomes [20]. Although the function of *AAD14*, a putative aryl-alcohol dehydrogenase, is not known, its introgression from *S. cerevisiae* into *S. paradoxus* [21] and its spread to five different *S. cerevisiae* chromosomes is suggestive of adaptation.

Methods: To test a gene for introgression, homologs, including orthologs and paralogs, were obtained from S288C, M22, YPS163, RM11, YJM789, *S. paradoxus*, *S. mikatae*, *S. bayanus* using

BLASTN. Homologs showing less than 80% identity at the nucleotide level were not considered. Homologs were aligned using Clustalw and maximum likelihood phylogenetic trees were obtained using Phylip. Heterogeneity in the tree topology was examined by sliding window analysis using Topali [22]. Introgressed genes were detected by cases where one or more *S. paradoxus* alleles grouped with one or more of the *S. cerevisiae* strains.

Ectopic Gene Conversion

If the shared polymorphism is the result of ectopic gene conversion, paralogs should be more similar to each other than their orthologs. To further test for gene conversion we constructed a maximum likelihood phylogenetic tree for the polyamine transporters *TPO2* and *TPO3*, which share two SNPs in regions of high homology (Dataset S1). Except for *S. bayanus*, for which only one gene was found, homologs from the same species are more similar to each other than they are to homologs from different species (Figure 4). Since the homologs are present at syntenic locations in each genome, it is unlikely that they were created by multiple recent duplication events. This implies that ectopic gene conversion has resulted in concerted evolution in *S. mikatae*, *S. paradoxus* and *S. cerevisiae* genomes. Given the strong evidence for ectopic gene conversion, elevated rates of polymorphism within duplicated sequences can in part be attributed to recent ectopic gene conversion events, as has been found in humans [23, 24].

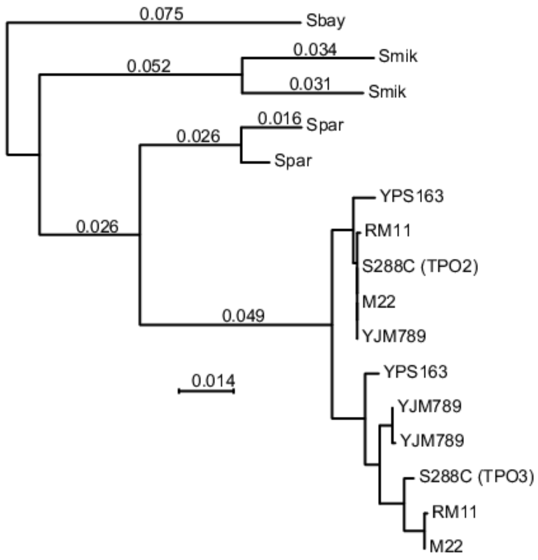

Figure 4. A maximum likelihood phylogeny supports concerted evolution of polyamine transporters. The phylogeny includes two polyamine transporters present in S288C, *TPO2* and *TPO3*. Two polyamine transporter homologous were identified in each genome and except for *S. bayanus* and YJM789, for which one and three homologous were identified, respectively.

Rates of Polymorphism Compared to Divergence

To compare rates of polymorphism and divergence, the genome of *S. paradoxus*, the closest

known relative of *S. cerevisiae*, was aligned to the S288C genome. Out of the 10.9 Mbp of aligned sequence, there is polymorphism data for 10.2 Mbp and data from all three strains for 7.6 Mbp, excluding InDels. To account for multiple substitutions, which can bias comparisons of polymorphic sites to fixed differences [25], we used maximum likelihood methods to estimate rates of polymorphism and divergence. Table S5 shows the rate of polymorphism relative to divergence across different classes of functional elements.

Using synonymous sites within coding sequences as a proxy for neutral sites, all classes show evidence of negative selection by their reduced rate of substitution between species. A number of classes of repetitive sequences show rates of polymorphism greater than that at synonymous sites. Although the coverage of telomeric sequences with polymorphism and divergence data is small, similarly high rates of polymorphism also occur in 9 kb of telomeric sequences for which there is only polymorphism data. The elevated rates of polymorphism in repetitive sequences is not due to negative selection on synonymous sites since total synonymous site diversity is slightly higher than diversity within low codon bias genes, defined by a codon adaption index [26] of less than 0.10. Rates of polymorphism could be influenced by ectopic gene conversion if recent since converted and non-converted alleles can remain segregating in the population [27]. The entire dataset is available in Supporting Information (Dataset S2).

Methods: Rates of polymorphism were estimated from the combined alignments across functional categories defined by S288C annotations from SGD. The rate of polymorphism was estimated for nine different functional classes defined in the SGD: autonomous replicating sequence (ARS), coding sequence (CDS), centromere (CEN), intron (INT), noncoding (NC), ncRNA (noncoding RNA), pseudogene (PG), repeat region (RR), transposable element (TE), transposable element gene (TEG), telomere (TEL), tRNA. For coding sequence, synonymous and nonsynonymous substitution rates were distinguished from one another. For each class, sequences containing both polymorphism and divergence data, gaps excluded, were concatenated and substitution rates were estimated by maximum likelihood using HyPhy [28], with a transition to transversion ratio of four, an HKY85 model [29] for all classes except coding regions, and an MG94 codon model combined with a HKY85 nucleotide model for coding regions [30]. Sequences for which there are overlapping coding annotations were excluded from the analysis.

McDonald-Kreitman Style Tests

The McDonald-Kreitman test [31] compares polymorphism and divergence at nonsynonymous to synonymous sites within coding sequences. This comparison largely accounts for variation in the genealogical history of a gene since synonymous and nonsynonymous sites are interspersed between one another. As originally formulated, the McDonald-Kreitman test does not account for differences in the rates of transitions and transversions and does not correct for multiple substitutions at the same site, both of which affect synonymous and nonsynonymous sites differently [32]. To account for these deficiencies and to accurately estimate differences in selective constraints on polymorphism and divergence data, we used maximum likelihood, implemented with the HyPhy software package [28].

For noncoding sequences variation in the genealogical history of a sample must be accounted for. To apply the McDonald-Kreitman test to noncoding sequences, we split each region into conserved and unconserved sites based on whether a site is conserved in the three-way alignment of *S. mikatae*, *S. bayanus* and *S. kudriavzevii*. Although this classification will not always distinguish functional from nonfunctional sites, this should not bias a McDonald-Kreitman test but may reduce its power. Based on this criterion, 51% of noncoding sites are classified as conserved and the average length of contiguously conserved and contiguously unconserved sites is 2.7 and 2.6 bp, respectively.

The three coding regions with the most significant excess of nonsynonymous polymorphism are *TCB3*, a lipid binding protein that functions in membrane trafficking [33], *OPT2*, an oligopeptide transporter, and *IRA2*, which negatively regulates the RAS-cAMP pathway [34]. The three noncoding regions with the most significant excess of polymorphism lie upstream of two uncharacterized genes and *YCP1*, an alkaline ceramidase that is involved in sphingolipid metabolism and confers resistance to the antifungal compound Fumonisine B1 [35].

Distinguishing Deleterious from Neutral SNPs

To identify deleterious noncoding SNPs, we also applied a likelihood ratio test to the small number of noncoding SNPs within sequences conserved to distantly related species. For noncoding sequences we compared the likelihood of three noncoding sites centered on the SNP for dNC equal to dS and dNC less than dS, where dNC is the noncoding substitution rate. We identified 309 deleterious noncoding SNPs (38% of those with distant homologs). However, the resolution of constraint within noncoding sequences is three bases and the likelihood ratio test may result in false positives when a SNP occurs in a neutral site flanked by two conserved sites.

The deleterious SNPs are not randomly distributed in the genome. Many of the deleterious noncoding SNP occur within repetitive elements (167), pseudogenes (21), tRNAs (7), autonomous replicating sequences (7), or noncoding RNA genes (5). Fifty-five of the 102 SNPs within sequences without any annotation occur within 50 bp of a tRNA gene. The distribution of the deleterious noncoding SNPs is likely a by-product of the ability to align these noncoding features across greater evolutionary distances (Figure 3) and suggests that many more deleterious noncoding SNPs would be identified if more noncoding sequences could be aligned beyond the sensu strict Saccharomyces species.

Across the 309 noncoding and 1472 coding SNPs, 49% occur in positions that are identical across all species and 21% occur in positions that vary but the derived SNP allele is not present in any of the other species. The remaining 30% of SNPs occur in sites where both alleles are present in at least one other species. The presence of the two alleles in other species can be explained by weak selection or lineage-specific constraints. Lineage-specific constraints can result from divergence in function or compensatory changes and have also been invoked to explain the observation that 10% of human disease alleles are present in other species [36].

Strength of Selection on Deleterious SNPs in Conserved Noncoding Motifs

To characterize the strength of selection against the deleterious SNPs, we estimated selection coefficients from the relative frequency of the two SNP alleles across all instances of a conserved motif. Although obtaining an accurate estimate of the strength of selection would be difficult, equilibrium frequencies provide a means of ranking the relative strength of selection, measured by $2Ns$, where N is the effective population size and s is the selection coefficient [37]. Of the 2452 SNPs, 691 (27%) have $2Ns$ values less than negative one. The estimated strength of selection may be biased towards zero as a result of including alignments evolving under a different set of selective constraints, e.g. spurious matches to the Phylonet motif.

Methods: The modified Phylonet algorithm produces a set of sequences that show constraints most similar to those adjacent to but not including the SNP position. To test whether the two SNP alleles are equivalent, we used a likelihood ratio test implemented in HyPhy [28] to compare two models. The null model assumes the equilibrium frequencies of the two SNP alleles are the same across all the alignments and the alternative model contains an additional parameter that allows for a

difference in the equilibrium frequency of the two alleles as measured by their relative fitness difference or selection coefficient. For cases where the base frequencies differed from the null model, we estimated the strength of selection by $2Ns = \ln(\pi_x/\pi_y)$, where π_x is the maximum likelihood estimate of the equilibrium frequency of the derived allele in all the instances of the conserved motif and π_y is the sum of all other equilibrium base frequencies.

LITERATURE CITED

1. Pop M, Phillippy A, Delcher AL, Salzberg SL (2004) Comparative genome assembly. Brief Bioinform 5: 237-248.
2. Liti G, Louis EJ (2005) Yeast evolution and comparative genomics. Annu Rev Microbiol 59: 135-153.
3. Lafontaine I, Fischer G, Talla E, Dujon B (2004) Gene relics in the genome of the yeast saccharomyces cerevisiae. Gene 335: 1-17.
4. McMahon ME, Stamenkovich D, Petes TD (1984) Tandemly arranged variant 5s ribosomal rna genes in the yeast saccharomyces cerevisiae. Nucleic Acids Res 12: 8001-8016.
5. Ronald J, Brem R, Whittle J, Kruglyak L (2005) Local regulatory variation in saccharomyces cerevisiae. PLoS Genet 1: e25.
6. Wei W, McCusker JH, Hyman RW, Jones T, Ning Y et al. (2007) Genome sequencing and comparative analysis of saccharomyces cerevisiae strain yjm789. Proc Natl Acad Sci U S A 104: 12825-12830.
7. Saccharomyces Genome Database (www.yeastgenome.org).
8. Shimoi H, Sakamoto K, Okuda M, Atthi R, Iwashita K et al. (2002) The awa1 gene is required for the foam-forming phenotype and cell surface hydrophobicity of sake yeast. Appl Environ Microbiol 68: 2018-2025.
9. Goto K, Iwatuki Y, Kitano K, Obata T, Hara S (1990) Cloning and nucleotide sequence of the khr killer gene of saccharomyces cerevisiae. Agric Biol Chem 54: 979-984.
10. Charron MJ, Dubin RA, Michels CA (1986) Structural and functional analysis of the mal1 locus of saccharomyces cerevisiae. Mol Cell Biol 6: 3891-3899.
11. Naumova ES, Naumov GI, Masneuf-Pomarède I, Aigle M, Dubourdieu D (2005) Molecular genetic study of introgression between saccharomyces bayanus and s. cerevisiae. Yeast 22: 1099-1115.
12. Lander ES, Waterman MS (1988) Genomic mapping by fingerprinting random clones: a mathematical analysis. Genomics 2: 231-239.
13. Ewing B, Green P (1998) Base-calling of automated sequencer traces using phred. ii. error probabilities. Genome Res 8: 186-194.
14. Walther D, Bartha G, Morris M (2001) Basecalling with lifetrace. Genome Res 11: 875-888.
15. Kim JM, Vanguri S, Boeke JD, Gabriel A, Voytas DF (1998) Transposable elements and genome organization: a comprehensive survey of retrotransposons revealed by the complete saccharomyces cerevisiae genome sequence. Genome Res 8: 464-478.
16. Entian KD, Schuster T, Hegemann JH, Becher D, Feldmann H et al. (1999) Functional analysis of 150 deletion mutants in saccharomyces cerevisiae by a systematic approach. Mol Gen Genet 262: 683-702.
17. Teunissen AW, Steensma HY (1995) Review: the dominant flocculation genes of saccharomyces

cerevisiae constitute a new subtelomeric gene family. Yeast 11: 1001-1013.
18. Garciadeblas B, Rubio F, Quintero FJ, Bañuelos MA, Haro R et al. (1993) Differential expression of two genes encoding isoforms of the atpase involved in sodium efflux in saccharomyces cerevisiae. Mol Gen Genet 236: 363-368.
19. Wieland J, Nitsche AM, Strayle J, Steiner H, Rudolph HK (1995) The pmr2 gene cluster encodes functionally distinct isoforms of a putative na+ pump in the yeast plasma membrane. EMBO J 14: 3870-3882.
20. Delneri D, Gardner DC, Oliver SG (1999) Analysis of the seven-member aad gene set demonstrates that genetic redundancy in yeast may be more apparent than real. Genetics 153: 1591-1600.
21. Liti G, Barton DBH, Louis EJ (2006) Sequence diversity, reproductive isolation and species concepts in saccharomyces. Genetics 174: 839-850.
22. Milne I, Wright F, Rowe G, Marshall DF, Husmeier D et al. (2004) Topali: software for automatic identification of recombinant sequences within dna multiple alignments. Bioinformatics 20: 1806-1807.
23. Jackson MS, Oliver K, Loveland J, Humphray S, Dunham I et al. (2005) Evidence for widespread reticulate evolution within human duplicons. Am J Hum Genet 77: 824-840.
24. Bailey JA, Eichler EE (2006) Primate segmental duplications: crucibles of evolution, diversity and disease. Nat Rev Genet 7: 552-564.
25. Templeton A (1996) Contingency tests of neutrality using intra/interspecific gene trees: the rejection of neutrality for the evolution of the mitochondrial cytochrome oxidase ii gene in the hominoid primates. Genetics 144: 1263-1270.
26. Sharp PM, Li WH (1987) The codon adaptation index--a measure of directional synonymous codon usage bias, and its potential applications. Nucleic Acids Res 15: 1281-1295.
27. Innan H (2003) A two-locus gene conversion model with selection and its application to the human rhce and rhd genes. Proc Natl Acad Sci U S A 100: 8793-8798.
28. Pond S, Frost S, Muse S (2005) Hyphy: hypothesis testing using phylogenies. Bioinformatics 21: 676-679.
29. Hasegawa M, Kishino H, Yano T (1985) Dating of the human-ape splitting by a molecular clock of mitochondrial dna. J Mol Evol 22: 160-174.
30. Muse SV, Gaut BS (1994) A likelihood approach for comparing synonymous and nonsynonymous nucleotide substitution rates, with application to the chloroplast genome. Mol Biol Evol 11: 715-724.
31. McDonald J, Kreitman M (1991) Adaptive protein evolution at the adh locus in drosophila. Nature 351: 652-654.
32. Hasegawa M, Cao Y, Yang Z (1998) Preponderance of slightly deleterious polymorphism in mitochondrial dna: nonsynonymous/synonymous rate ratio is much higher within species than between species. Mol Biol Evol 15: 1499-1505.
33. Creutz CE, Snyder SL, Schulz TA (2004) Characterization of the yeast tricalbins: membrane-bound multi-c2-domain proteins that form complexes involved in membrane trafficking. Cell Mol Life Sci 61: 1208-1220.
34. Tanaka K, Nakafuku M, Satoh T, Marshall MS, Gibbs JB et al. (1990) S. cerevisiae genes ira1 and ira2 encode proteins that may be functionally equivalent to mammalian ras gtpase activating protein. Cell 60: 803-807.
35. Mao C, Xu R, Bielawska A, Obeid LM (2000) Cloning of an alkaline ceramidase from saccharomyces cerevisiae. an enzyme with reverse (coa-independent) ceramide synthase activity. J Biol Chem 275: 6876-6884.

36. Kondrashov A, Sunyaev S, Kondrashov F (2002) Dobzhansky-muller incompatibilities in protein evolution. Proc Natl Acad Sci U S A 99: 14878-14883.
37. Bulmer M (1991) The selection-mutation-drift theory of synonymous codon usage. Genetics 129: 897-907.
